# High-throughput field phenotyping reveals that selection in breeding has affected the phenology and temperature response of wheat in the stem elongation phase

**DOI:** 10.1093/jxb/erad481

**Published:** 2023-12-22

**Authors:** Lukas Roth, Lukas Kronenberg, Helge Aasen, Achim Walter, Jens Hartung, Fred van Eeuwijk, Hans-Peter Piepho, Andreas Hund

**Affiliations:** ETH Zurich, Institute of Agricultural Sciences, Universitätstrasse 2, 8092 Zurich, Switzerland; ETH Zurich, Institute of Agricultural Sciences, Universitätstrasse 2, 8092 Zurich, Switzerland; ETH Zurich, Institute of Agricultural Sciences, Universitätstrasse 2, 8092 Zurich, Switzerland; Agroscope, Earth Observation of Agroecosystems Team, Division Agroecology and Environment, Reckenholzstrasse 191, 8046 Zurich, Switzerland; ETH Zurich, Institute of Agricultural Sciences, Universitätstrasse 2, 8092 Zurich, Switzerland; University of Hohenheim, Institute for Crop Science, Biostatistics Unit, Fruwirthstrasse 23, D-70593 Stuttgart, Germany; Wageningen University and Research, Biometris, PO Box 16, 6700 AA Wageningen, The Netherlands; University of Hohenheim, Institute for Crop Science, Biostatistics Unit, Fruwirthstrasse 23, D-70593 Stuttgart, Germany; ETH Zurich, Institute of Agricultural Sciences, Universitätstrasse 2, 8092 Zurich, Switzerland; CIMMYT, Mexico

**Keywords:** Correlated response to selection, genetic correlation, genomic prediction, growth dynamic, GWAS, modeling, trait extraction

## Abstract

Crop growth and phenology are driven by seasonal changes in environmental variables, with temperature as one important factor. However, knowledge about genotype-specific temperature response and its influence on phenology is limited. Such information is fundamental to improve crop models and adapt selection strategies. We measured the increase in height of 352 European winter wheat varieties in 4 years to quantify phenology, and fitted an asymptotic temperature response model. The model used hourly fluctuations in temperature to parameterize the base temperature (*T*_min_), the temperature optimum (*r*_max_), and the steepness (lrc) of growth responses. Our results show that higher *T*_min_ and lrc relate to an earlier start and end of stem elongation. A higher *r*_max_ relates to an increased final height. Both final height and *r*_max_ decreased for varieties originating from the continental east of Europe towards the maritime west. A genome-wide association study (GWAS) indicated a quantitative inheritance and a large degree of independence among loci. Nevertheless, genomic prediction accuracies (GBLUPs) for *T*_min_ and lrc were low (*r*≤0.32) compared with other traits (*r*≥0.59). As well as known, major genes related to vernalization, photoperiod, or dwarfing, the GWAS indicated additional, as yet unknown loci that dominate the temperature response.

## Teaser

Wheat phenology and environmental responses have been affected by breeders’ selections since 1970. High-throughput field phenotyping methods reveal these developments, allowing better adaptation of future varieties to climate change.

## Introduction

Mitigating climate change impacts on crops through genotypic adaptation requires understanding crop responses to environmental factors (Ramirez-Villegas *et al.*, 2015). Responses of major crops are well studied in controlled environments but the translation of insights to the field is not straightforward (Poorter *et al.*, 2016). High-throughput field phenotyping (HTFP) may facilitate this transition (Araus *et al.*, 2018).

A main driver of plant growth and development is temperature (Porter and Gawith, 1999). Examining the influence of breeding on the temperature response is challenging: in 17 crop species including wheat (*Triticum aestivum* L.), Parent and Tardieu (2012) found no indications of such relationships using mainly short-term experiments conducted under controlled conditions. In contrast, Kronenberg *et al.* (2020a) found a genotype-specific temperature response for a set of European winter wheat genotypes in the field (the GABI-Wheat panel, Kollers *et al.*, 2013; Gogna *et al.*, 2022). A crucial difference between the two investigations is that the former normalized growth rates of genotypes to unity at 20 °C while the latter did not. Thus, Kronenberg *et al.* (2020a) have used an approach that allowed genotypes to differ in growth rates at optimal temperature. In addition, the ~300 GABI-Wheat panel genotypes represent a wide European diversity while the seven genotypes examined in Parent and Tardieu (2012) are mainly from Australia.

Gaining insights on adverse aspects of temperature response and phenology (i.e. the timing of key stages) is of high interest for breeding. Investigations of historic US corn belt data, for example, indicated an indirect selection for temperature response in commercial maize hybrids (Kusmec *et al.*, 2023). Increasing the duration of stem elongation by adjusting heading time or the beginning of stem elongation (jointing) has repeatedly been proposed as a possibility to increase wheat yield (Slafer *et al.*, 1996; Miralles and Slafer, 2007). Phenology is driven by environmental (E) and genotype (G) characteristics and corresponding interactions, and therefore is a result of G×E. In contrast, temperature response traits are only to a limited extent affected by—but are rather drivers of—G×E (Roth *et al.*, 2022b, 2023). Describing such responses directly in the breeding nursery may allow breeders to predict the phenotypic performance in new unseen environments (Poorter *et al.*, 2010). Yet, differences in the development of wheat varieties originating from various world regions are not well documented and understood (Gbegbelegbe *et al.*, 2017).

With the advent of high-throughput phenotyping methods, the characterization of large genotype panels has become feasible. One of the most simple traits to detect is plant height. This trait can be analyzed at a temporal resolution of a few days with several methods. Kronenberg *et al.* (2020a) used a terrestrial laser scanner mounted on a rope-suspended phenotyping platform (Kirchgessner *et al.*, 2017) to determine plant height. From a breeder’s perspective, such a stationary platform is highly inflexible as it does not allow screening of multi-environment trials. Mobile platforms are better suited to screen large breeding populations (Aasen and Bareth, 2018), allowing the genetic gain of selection to be increased (Araus *et al.*, 2018). Thus, the first aim of this study was to test the suitability of a drone-based phenotyping platform carrying RGB-cameras (Roth *et al.*, 2018) as a replacement for laser scanning-based phenotyping (Kronenberg *et al.*, 2020a).

Independent of the measurement device, modeling the temperature response from field-derived data bears flaws and pitfalls (Roth *et al.*, 2022b). The eligibility of a temperature response curve (i.e. a dose–response curve) does not depend only on the (biological) response but also on the range of measured temperatures. Using a linear regression to model temperature response as done in Kronenberg *et al.* (2020a) is controversial: such a Type 1 response (Wang *et al.*, 2017) will come to its limits when measurements span a whole growing season with temperatures also extending into supra-optimal ranges (Parent *et al.*, 2019; Kronenberg *et al.*, 2020a). Using an asymptotic temperature response curve may overcome this limitation and allows resolution of the temperature response into a base temperature (*T*_min_), the growth rate at optimal temperatures (*r*_max_), and the steepness of the temperature response (lrc) (Roth *et al.*, 2022b). Phenology-related traits can be extracted from the same height time series using, for example, a spline approach (Roth *et al.*, 2021). Having first assessed the quality of drone-based height data, the second aim of this study was to evaluate whether the asymptotic model and the spline approach are suitable to extract meaningful parameters from such data.

With the advent of global climate change, the adjustment of phenology was and is a major breeding aim. The hereafter examined GABI-Wheat panel (Kollers *et al.*, 2013; Gogna *et al.*, 2022) includes important genotypes from different climatic regions of Europe. European countries have pursued country-specific, largely independent breeding programs. A certain degree of population structures may have arisen from a co-selection of different alleles based on country-specific climate constraints and management practices (Zanke *et al.*, 2014). On the other hand, phenology and other traits may also be genetically linked *per se*—showing pleiotropic effects. While genetic correlations can be used to analyze the correlated response of traits to selection (Falconer and Mackay, 1996), genome-wide association studies (GWAS) allow insights into the genetic architecture of the investigated traits as well as their inter-relationships to be obtained.

Hence, after evaluating the suitability of drone data to extract phenology and temperature response traits, the third aim of this study was to characterize the GABI-Wheat panel using phenotypic and genetic correlations and GWAS, providing insights on direct and indirect response to selection in breeding programs but also on general genetic relationships.

## Materials and methods

### Experimental design

Experiments were performed in 4 consecutive years (2015–2018) in the field phenotyping platform FIP (Kirchgessner *et al.*, 2017) at the ETH research station of agricultural sciences in Lindau Eschikon, Switzerland (47.449 N, 8.682 E, 556 m a.s.l.). Details about designs, genotypes, soil, and management can be found in Kronenberg *et al.* (2017, 2020a) for 2015–2017 and in Roth *et al.* (2020) for 2018.

In brief, a GABI-Wheat subset (consisting of 300 European winter wheat cultivars from the GABI-Wheat panel, Kollers *et al.*, 2013; Gogna *et al.*, 2022) was complemented by 35–52 Swiss winter wheat varieties of commercial importance. The resulting panel of, on average, 345 genotypes was replicated twice per year and each replication was planted on a different lot in the FIP area. Each replication was augmented with checks in a 3 × 3 block arrangement (Supplementary Fig. S1). Designs were enriched with spatial coordinates based on unmanned aerial system (UAS) flights for the years 2017 and 2018. For 2015 and 2016, no UAS flights were available and therefore local coordinates (row and range) were used as the spatial context, as described by Kronenberg *et al.* (2020a).

### Phenotyping and covariate measurements

Plant height measurements for the years 2015–2017 were taken using a terrestrial laser scanner (TLS) based on a light detection and ranging (LiDAR) sensor (FARO R Focus3D S 120; Faro 113 Technologies Inc., Lake Mary, FL, USA) mounted on the FIP (Kronenberg *et al.*, 2017). Measurements were performed every 3–4 d from jointing to harvest in 2015 and 2016, and from tiller development to harvest in 2017 (Kronenberg *et al.*, 2020a).

For the year 2018, FIP measurements were replaced by the UAS platform PhenoFly (Roth *et al.*, 2018). The UAS captured RGB images with high spatial overlap that were processed using Structure-from-Motion (SfM) software (Agisoft Metashape, Agisoft LLC, St. Petersburg, Russia) to yield digital plant height models. General flight campaign settings are described in Roth *et al.* (2020). Flights were performed every 2–3 d from tiller development to harvest.

To ensure comparability between TLS and UAS data, measurements were performed with both platforms simultaneously on five dates in 2018 (April 6, 11, and 19, and May 9 and 14) for one replication. The number of replications was reduced to one in order to reduce the data collection effort. Replicates allow the calculation of heritabilities and adjusted genotype means to compensate for, for example, spatial gradients. For comparing plot measurements, this is not required, as one aims at comparing (unadjusted) phenotype values.

Meteorological data were obtained from a weather station next to the experimental field (50 m). Data gaps were filled with data from a nearby public Agrometeo weather station (http://www.agrometeo.ch/, Agroscope, Nyon, Switzerland) in proximity (550 m).

### Plant height extraction

For TLS measurements, individual plot-based plant height values were extracted from point clouds using a custom-developed Matlab script as described in Friedli *et al.* (2016) and Kronenberg *et al.* (2017). For UAS measurements, plot-based values were extracted in Python as described by Roth and Streit (2018) with one modification: before processing digital elevation models (DEMs) to plant height models, DEMs were spatially corrected using reference ground control point (GCP) coordinates. To do so, differences between DEM elevations and known reference elevations were calculated at all GCP locations, and a cubic interpolation was performed on the whole experimental area. Interpolated differences were then subtracted from the original DEM to produce a corrected DEM.

### Dynamic modeling

#### Timing of jointing, end of stem elongation, and final height

**Fig. 1.**
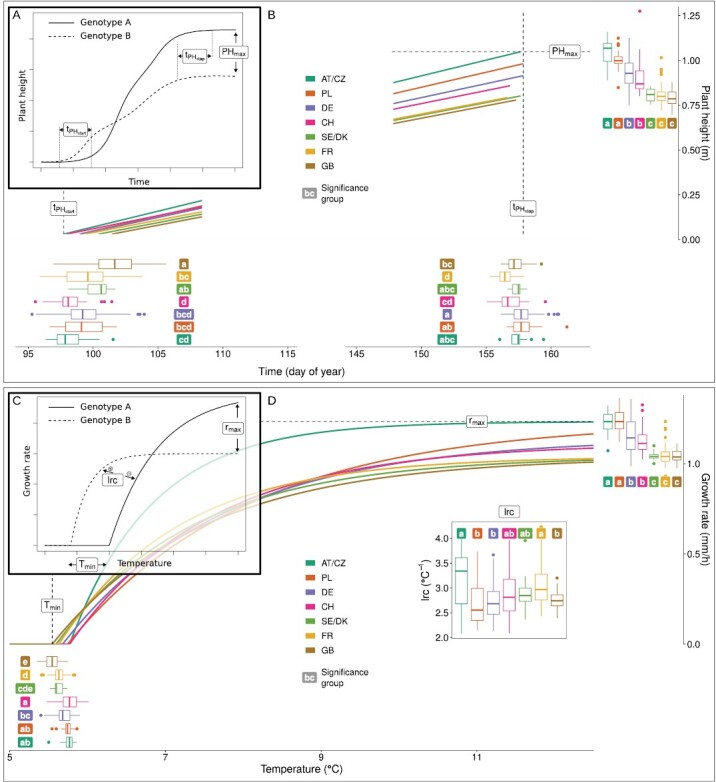
Schematic representation of derived traits (A, C) and real data trends in selection within country of registration groups (B, D). Traits were derived from the spline models followed by the quarter of maximum elongation rate (QMER) method based on extraction of timing of key stage traits jointing ($t_{PH_{start}}$) and end of stem elongation ($t_{PH_{stop}}$) and quantity trait final height (PH_max_) (A) and from the asymptotic model to determine the temperature response parameters maximum elongation rate (*r*_max_), base temperature where the elongation rate is zero (*T*_min_), and steepness of the response (lrc) (C). Box plots (B, D) indicate the distribution of genotypes within country groups, and solid colored lines indicate the group medians. Significant (α=0.05) differences between country groups are indicated by letters (a–e; country groups not sharing a letter are significantly different).

In a first step, a shape-constrained monotonically increasing P-spline was fitted to plot time series. Then, the quarter of maximum elongation rate (QMER) method (Roth *et al.*, 2021) was applied to extract the growth stages jointing ($t_{PH_{start}}$) and end of stem elongation ($t_{PH_{stop}}$). In brief, the QMER method determines the time point at which the elongation rate exceeds $t_{PH_{start}}$ or falls short of $t_{PH_{stop}}$ by a certain threshold of the maximum elongation rate. The threshold was determined based on a wheat growth simulation (Roth *et al.*, 2021) and empirical data for wheat (Roth *et al.*, 2023) and soybean (Roth *et al.*, 2022a) to 1/4, thus named ‘quarter of maximum elongation rate’ (QMER). Final height (PH_max_) was calculated as the median of the top 24 spline predictions after the estimated stop of growth $t_{PH_{stop}}$ (Fig. 1A). Further details on how to extract such timing and quantity traits are described in Roth *et al.* (2021).

#### Temperature dose–response parameters

Measuring plant height with high-throughput devices allows growth rates to be derived from successive measurements (Kronenberg *et al.*, 2020a). To extract the temperature response of growth from these time series, an asymptotic model (Fig. 1C) was fitted to hourly temperature values and irregular height measurements using maximum-likelihood fitting as described in Roth *et al.* (2021). The model extracts the parameters *r*_max_ (the maximum elongation rate and therefore the asymptote of the curve), *T*_min_ (the base temperature where the elongation rate is zero), and lrc (the steepness of the response).

To allow for a comparison with previous field-based studies (Grieder *et al.*, 2015; Kronenberg *et al.*, 2020a), an additional linear model was fitted to the data. This model regressed growth rates on mean air temperatures of the corresponding measurement period (i.e. the mean of a 3–5 d hourly temperature time series), thus extracting the temperature response parameter *lm*_slope_ which corresponds to the slope reported in Kronenberg *et al.* (2020a).

### Adjusted genotype means per year and repeatability

The above-described dynamic modeling of plot-based repeated measures into plot-based intermediate traits can be seen as a first stage of stage-wise processing (Roth *et al.*, 2021). These plot-based intermediate traits were further processed in a stage-wise weighted linear mixed model analysis (stage two and three), in which the second stage averaged over within-year effects (resulting in adjusted genotype-year means) and the third stage over between-year effects (resulting in overall adjusted genotype means). For the second stage, the R package SpATS (Rodríguez-Álvarez *et al.*, 2018) was parameterized with a mixed model with spatial components as described in Roth *et al.* (2021).

Weighting was only applied when decreasing the Bayesian information criterion (BIC) if compared with a model without weighting. Weights did not improve BIC for 2018 for *r*_max_, for all years for *T*_min_, for 2015–2017 for lrc, for 2017 for $t_{PH_{start}}$, and for 2015, 2016, and 2017 for $t_{PH_{stop}}$. Within-year heritability (repeatability, *H*^2^_*j*_) based on BLUPs was calculated according to Oakey *et al.* (2006).

### Across-year adjusted genotype means and heritability

For the third stage, the R package ASReml-R (Butler, 2018) was parametrized with the model


$$\begin{array}{l} {\hat{\theta }}_{ij}=\mu +u_{j}+\theta_{i}+{\left(\theta u\right)}_{ij}+e_{ij}, \end{array}$$ (1)

where ${\hat{\theta }}_{ij}$ are adjusted year genotype means (BLUE) from the previous stage, μ is a global intercept, *u*_*j*_ represents year effects, θ_*i*_ genotype responses, (θ*u*)_*ij*_ genotype–year interactions allowing for year-specific variances (diagonal variance structure), and *e*_*ij*_ residuals weighted based on the inverse of the diagonal of the variance–covariance matrix from the previous stage.

For BLUEs calculations, μ and θ_*i*_ were set as fixed, and all other terms as random. For BLUPs and heritability calculations, θ_*i*_ was set as random with known variance structure based on the normalized genome-wide average identity by state (IBS) relationship structure calculated from single nucleotide polymorphism (SNP) marker data using the snpgdsIBS function in the R-package SNPRelate (Zheng *et al.*, 2012).

Marker data were supplied by the GABI wheat consortium (Kollers *et al.*, 2013; Gogna *et al.*, 2022) for the GABI wheat genotypes and by Agroscope in the framework of the Swiss winter wheat breeding program (Fossati and Brabant, 2003) for the Swiss genotypes. For the IBS analysis, only non-monomorphic SNPs with unequivocal genome positions (see Kronenberg *et al.*, 2020a), a missing rate <0.05, and a minor allele frequency <0.05 were used, thus resulting in 9147 SNPs for 325 genotypes. Chromosome-specific distance thresholds of linkage disequilibrium (LD) decay below *R*^2^=0.2 were calculated as described in Kronenberg *et al.* (2020a). Heritability was calculated on a genotype difference basis following the *H*^2^_ΔBLUP_ method defined in Schmidt *et al.* (2019).

### Phenotypic and genetic correlation calculation

The phenotypic correlations between traits were calculated for each of the 4 examined years as Pearson’s *r* of plot-based values. For reporting, the mean, maximum, and minimum of these four correlations per trait pair were calculated. For the genetic correlation calculation, the univariate model of Equation 1 was extended to a bivariate model (Wright, 1998; Holland *et al.*, 2001),


$$\begin{array}{l} \left(\begin{array}{l} {\hat{\theta }}_{ij}^{t1}\;{\hat{\theta }}_{ij}^{t2}\; \end{array}\right)=\left(\begin{array}{l} \mu^{t1}\;\mu^{t2}\; \end{array}\right)+\left(\begin{array}{l} u_{j}^{t1}\;u_{j}^{t2}\; \end{array}\right)+\left(\begin{array}{l} \theta_{i}^{t1}\;\theta_{i}^{t2}\; \end{array}\right)+\left(\begin{array}{l} {\left(u\theta \right)}_{ij}^{t1}\;{\left(u\theta \right)}_{ij}^{t2}\; \end{array}\right)+\left(\begin{array}{l} e_{ij}^{t1}\;e_{ij}^{t2}\; \end{array}\right), \end{array}$$ (2)

where ${\hat{\theta }}_{ij}^{t1}$ and ${\hat{\theta }}_{ij}^{t2}$ are adjusted year genotype means (BLUEs) per trait [trait 1 (*t*1) and trait 2 (*t*2)] from the second stage, μ^*t*1^ and μ^*t*2^ are global intercepts per trait, $u_{j}^{t1}$ and $u_{j}^{t2}$ are year effects per trait, $\theta_{i}^{t1}$ and $\theta_{i}^{t2}$ are genotype responses (with known variance structure based on IBS), and ${\left(u\theta^{t1}\right)}_{ij}$ and ${\left(u\theta^{t2}\right)}_{ij}$ are the genotype responses to year interactions with uniform variances per trait. The terms μ^*t*1^, μ^*t*2^, $u_{j}^{t1}$ and $u_{j}^{t2}$ were set as fixed, and all other terms were random. Note that no variance–covariance matrix from the previous stage for a bivariate model was available. Consequently, *e* and (*u*θ) are confounded, and so the two terms were summarized in one variance–covariance structure. Genetic correlations among traits were then calculated based on the estimated variance and covariance components (Holland *et al.*, 2001),


$$\begin{array}{l} Corr\left(\theta^{t1}\;,\theta^{t2}\right)=\frac{Cov\left(\theta^{t1}\theta^{t2}\right)}{\sqrt{Var\left(\theta^{t1}\right)}\sqrt{Var\left(\theta^{t2}\right)}}. \end{array}$$ (3)

To investigate the effect of release year and country of origin on intermediate traits, the year and country of first registration of genotypes were looked up in the EU plant variety database (https://ec.europa.eu/food/plant/plant_propagation_material/plant_variety_catalogues_databases). Five multi-year groups ((1970, 1990], (1990, 1995], (1995, 2000], (2000, 2005], and (2005, 2018]) and seven countries groups (AT/CZ, PL, DE, CH, SE/DK, FR, and UK) were chosen, and phenotypic values per group were aggregated to means and SEs. Note that only country groups with a sample size ≥10 were considered, and that the first and last year clusters have, due to the focus of the GABI-Wheat panel on the release years 1990–2005, wider ranges than the other clusters.

### Genomic prediction and genome-wide association studies

In a next step, the suitability of the extracted intermediate traits for genomic prediction was estimated. To align results with the existing literature (e.g. Bustos-Korts *et al.*, 2019; Meher *et al.*, 2022; Toda *et al.*, 2022), overall genotype means (${\hat{\theta }}_{i}$ in Equation 1) were used as phenotypic values.

The prediction accuracy was evaluated based on a genomic best linear unbiased prediction (GBLUP) model parametrized in the R package ASReml-R (Butler, 2018),


$$\begin{array}{l} {\hat{\theta }}_{i}=\mu +G_{i}+e_{i}, \end{array}$$ (4)

where ${\hat{\theta }}_{i}$ are across-year BLUEs (Equation 1), μ a global intercept, and *G*_*i*_ are random genotype effects with *G*=(*G*_1_,*G*_2_,…)^*T*^ based on a variance–covariance matrix calculated as the IBS relationship structure mentioned above. Residuals *e*_i_ were weighted based on the inverse of the diagonal of the variance–covariance matrix from the previous stage (Equation 1). Prediction accuracy was calculated as mean Pearson’s *r* of 10-fold cross-validations; random folds were repeated 10 times.

To investigate the underlying genetic architecture of the different traits and assess the observed phenotypic and genetic correlations in this context, we performed GWAS on the 325 wheat varieties present across all year–sites. The same genotype data were used as for the IBS analysis (see above). Univariate GWAS were conducted for all traits using three different models implemented in the R package GAPIT3 (v. 3.1.0) (Wang and Zhang, 2021). As a baseline approach, a single locus, compressed, mixed linear model (MLM) (Zhang *et al.*, 2010) was used including the first three principal components (PCs) of the marker genotypes as fixed effects and a kinship matrix calculated following VanRaden (2008) as random effects for all traits. Further, the two multi-locus models FarmCPU (Liu *et al.*, 2012) and Blink (Huang *et al.*, 2018) were applied. The number of PCs was chosen based on visual inspections of the scree-plot and variance explained. For FarmCPU and Blink, PCs were omitted for all traits as the respective quantile–quantile plots showed better quality in the models without PCs compared with three PCs (Supplementary Fig. S2). While MLM effectively controls type I errors, the incorporated kinship and population structure can reduce the detection of true associations, especially for complex traits associated with population structure (Atwell *et al*, 2010; Liu *et al.*, 2012). FarmCPU and Blink have increased power as they reduce this confounding while maintaining the control of type I errors of MLM (Liu *et al.*, 2012; Huang *et al.*, 2018).

In order to investigate putative pleiotropic structures among the observed traits and account for the correlation structure between traits, we conducted multivariate GWAS using the software GEMMA (Zhou and Stephens, 2014). To this end, traits (excluding *lm*_slope_) were grouped into two physiological and correlation-based multi-trait combinations: (i) temperature response traits (*r*_max_, *T*_min_, lrc) and final height; and (ii) phenology traits ($t_{PH_{start}}$ and $t_{PH_{stop}}$) and final height. For all multivariate GWAS, the first three PCs were included to correct for population structure, and the same IBS matrix as used for genetic correlations was applied to correct for relatedness.

All GWAS were conducted on adjusted genotype means per year–site (BLUEs), as well as for across-year adjusted genotype means (BLUEs and BLUPs). For the detection of significant marker–trait associations (MTAs), a Bonferroni threshold [α=0.05, –log10(*P*)=5.26] was applied to stringently correct for multiple testing.

## Results

### Plant height measurements reveal characteristics of growth dynamics

**Fig. 2.**
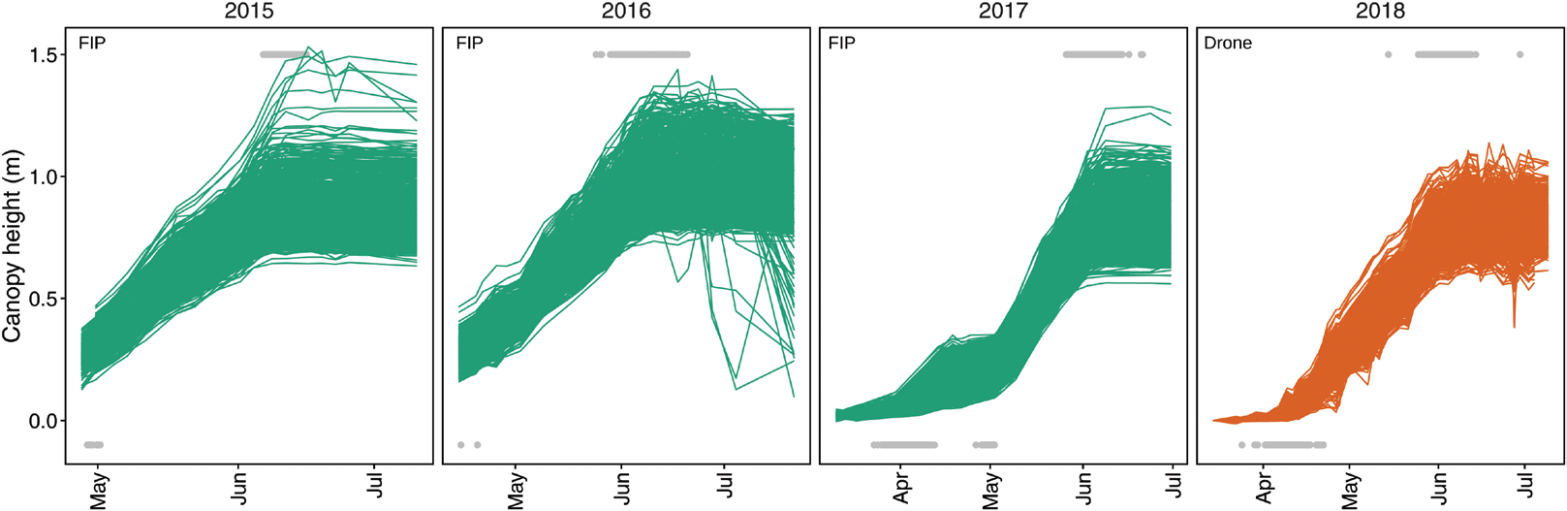
Plant height measurements performed with the field phenotyping platform (FIP, green) using a terrestrial laser scanner and with the PhenoFly platform (UAS, orange) using Structure-from-Motion based on RGB images. Gray points indicate detected start (bottom row) and end (top row) points of growth phases ($t_{PH_{start}}$ and $t_{PH_{stop}}$) (note that $t_{PH_{start}}$for 2015 and 2016 was not reliably detected, thus 2015 and 2016 $t_{PH_{start}}$values were skipped for subsequent analyses).

A total of 72 278 plant height estimation data points were extracted from TLS point clouds and UAS-based digital elevation models, corresponding to 2936 plot-based time series (Fig. 2). These plant height time series exhibited a strong increase after the start phase in the early season, and a clear plateau after reaching the maximum height mid-season. In 2016, time series indicated lodging for specific plots at the end of this extraordinarily wet growing season. The start phase of stem elongation exhibited a clear lag in the second half of April 2017, but not for other years. The dynamics of the end phase of stem elongation visually did not differ between years. Final heights clearly differed between years, with tall plants in the wet year 2016 and short plants in the extraordinarily dry year 2018.

**Fig. 3.**
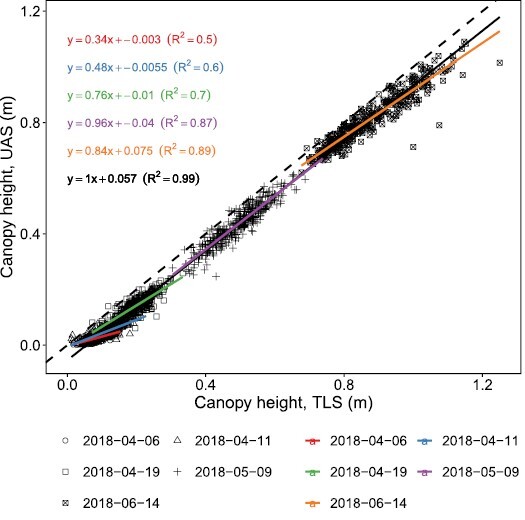
Comparison of terrestrial laser scanning- (TLS) based plant heights and drone- (UAS) based plant heights for five measurement dates in 2018. Colored lines are linear regressions for corresponding dates, the black line is a linear regression for all dates, the colored and black text lines denote the slope, intercept, and goodness of fit (*R*^2^) for the corresponding linear regressions, and the dashed line annotates a 1:1 relationship.

TLS and UAS measurements performed in parallel in 2018 revealed good correlations with moderate coefficients of determination for three early dates (*R*^2^=0.5–0.7) and strong coefficients of determination for two later dates (*R*^2^=0.87–0.89) (Fig. 3). Intercepts of the first two dates were close to zero, negative for the two subsequent dates (–0.01 m, –0.04 m), and positive for the last date (0.075 m); slopes indicated a severe underestimation of height by UAS measurements for early dates but weaker underestimation for later dates.

### Dynamic modeling allows extracting heritable timing of jointing, end of stem elongation, and final height traits

Fitted P-splines indicated a clear plateau after reaching final height ($t_{PH_{stop}}$/PH_max_) (Supplementary Fig. 3B). Visualizing the first derivative of the splines revealed a non-steady growth phase with severe changes in growth rates (Supplementary Fig. S3A). Applying the QMER method to determine the timing of jointing and end of stem elongation ($t_{PH_{start}}$ and $t_{PH_{stop}}$) to these non-steady growth phases led to visually coherent results (Supplementary Fig. S3A, B, vertical lines). Nevertheless, extracting the timing of jointing was only possible for the years 2017 and 2018 when early measurements before jointing were available. These early measurements are essential for the QMER method to determine the time point at which the growth rate first exceeds a certain threshold. In contrast, the end of stem elongation and final height could be extracted for all years, as late measurements after the stop of stem elongation were available.

Repeatabilities for year-specific adjusted genotype means were close to 1.0 for final height, and >0.68 for the timing of jointing and the end of stem elongation (Table 1). The heritability of across-year adjusted genotype means was highest for PH_max_ (0.98), followed by $t_{PH_{stop}}$ (0.87) and $t_{PH_{start}}$ (0.77). The genomic prediction accuracy for final height was, at 0.78, superior to all other traits. For the timing traits, the prediction accuracies were, at 0.59–0.61, lower but still strong.

**Table 1.** Repeatabilities (*H*^2^_*j*_), heritabilities (*H*^2^_ΔBLUP_), and genomic prediction accuracies (*r*, variance in parentheses) for extracted parameters and growing seasons

	2015	2016	2017	2018	All	All
Parameter	*H* ^2^_*j*_	*H* ^2^_*j*_	*H* ^2^_*j*_	*H* ^2^_*j*_	*H* ^2^_ΔBLUP_	*r* (var)
$t_{PH_{start}}$	–	–	0.68	0.74	0.77	0.61 (0.010)
$t_{PH_{stop}}$	0.77	0.86	0.83	0.71	0.87	0.59 (0.013)
PH_max_	0.98	0.99	0.98	0.97	0.98	0.78 (0.004)
*r*_max_	0.78	0.78	0.74	0.67	0.89	0.71 (0.007)
*T*_min_	0.33	0.23	0.36	0.32	0.29	0.18 (0.022)
lrc	0.57	0.21	0.52	0.75	0.63	0.32 (0.022)
*lm*_slope_	0.74	0.90	0.88	0.37	0.78	0.71 (0.008)

### Temperature dose–response modeling allows extracting heritable curve parameters

Fitting the asymptotic model produced dose–response curves with a distinct base temperature (*T*_min_) (Supplementary Fig. S3C). The increase (lrc) in elongation rate between the base temperature and asymptote was very steep for some plots in 2016 and 2017 (e.g. plot ‘FPWW0120005’ and ‘FPWW0180013’ in Supplementary Fig. S3C) but very flat for others in 2015 and 2018 (e.g. plot ‘FPWW0070043’ and ‘FPWW0220044’ in Supplementary Fig. S3C).

For the parameter *r*_max_, indicating the maximum elongation rate, the year-specific repeatabilities were generally high, but were lower for the years 2017 and 2018 than for other years (Table 1). High temperatures were more frequent in the years 2017 and 2018 compared with 2015 and 2016 (Supplementary Fig. S3D).

For the parameter *T*_min_, indicating the base temperature of growth, year-specific repeatabilties were very low for the year 2016, and higher for other years, with the highest value for 2017 (Table 1). Temperatures below zero were frequent for the year 2017 (with extremely low temperatures at the end of April) but less frequent for other years.

For the parameter lrc, indicating the steepness of growth response to temperature between the base temperature and maximum elongation rate, year-specific repeatabilities were very low for the year 2016 but higher for other years, with the highest value for 2018. The linear temperature response parameter *lm*_slope_ that incorporates both response to temperature and growth at optimum temperature had a very high across-year heritability. Nevertheless, it showed a large variation in repeatability between years, with a very low value (0.37) for the 2018 season.

In general, repeatabilities revealed large variations for temperature dose–response curve parameters among years (Table 1). Nonetheless, the across-year heritability was >0.63 for the two traits *r*_max_ and lrc, indicating a strong physiological basis. This finding was further confirmed by the strong genomic prediction accuracy of 0.71 for *r*_max_. Nevertheless, the prediction accuracies of *T*_min_ and lrc were lower (0.18–0.32).

### Grouping by country of registration reveals trends of selection

Significant effects of registration country were observed for the two phenology traits, all temperature response parameters, and PH_max_ (Fig. 1B, D). The genotype means for PH_max_ (final height) and *r*_max_ (growth rate at optimum temperature) showed the same pattern of AT/CZ≥PL>DE≥CH>SE/DK≥FR≥UK, and consequently the largest differences for PH_max_ (0.28 m) and *r*_max_ (0.20 mm h^–1^) were found between AT/CZ and UK.

In comparison, the pattern for $t_{PH_{start}}$ (jointing) was roughly inverted, with AT/CZ≤CH≤PL≤DE≤FR≤SE/DK≤UK. Again, the largest difference was found between early AT/CZ and late UK genotypes (3.8 d). The CH genotypes were also early, only 0.2 d later than AT/CZ.

The steepness of temperature response parameter lrc showed a pattern with PL and AT/CZ on the extremes, PL≤DE≤UK≤SE/DK≤CH≤FR≤AT/CZ. Consequently, AT/CZ genotypes exhibited a significantly steeper response to temperature (3.3 °C^–1^) than PL genotypes (2.6 °C^–1^). The genotype means for $t_{PH_{stop}}$ (end of stem elongation) showed a comparable pattern: FR genotypes were the earliest and PL and DE genotypes the latest, with the largest difference (1.3 d) between FR and DE. Finally, the temperature response parameter *T*_min_ (minimum temperature of growth) was related to PH_max_ and *r*_max_, with UK genotypes among those with the lowest *T*_min_ (mean: 5.5 °C) and AT/CZ among those with the highest *T*_min_ (mean: 5.8 °C).

Thus, varieties registered in UK were the latest to start jointing and had the lowest minimum temperature of growth, while CH genotypes were among the earliest to start jointing and showed, together with AT/CZ varieties, the highest minimum temperature of growth.

**Fig. 4.**
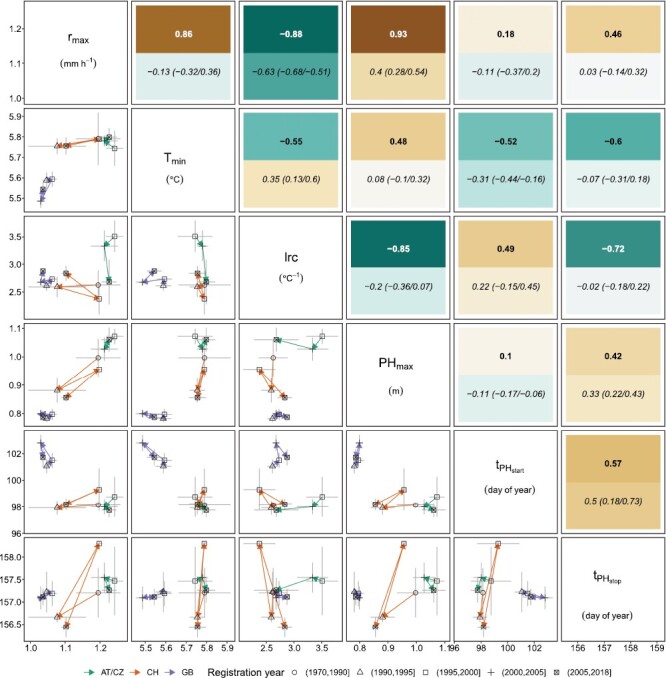
Genetic trait correlations for all 352 genotypes (upper triangle, bold), phenotypic correlations for all 352 genotypes (upper triangle, italic, mean value with minimum and maximum values indicated in parentheses), and mean and SE for genotype BLUPs of AT/CZ, CH, and UK genotype (lower triangle; see color key). The country-specific vector paths (arrows) represent the temporal development of the trait dependent on the registration year (see symbol key). All correlations are significant ((α=0.05) unless otherwise indicated (ns).

When visualizing the temporal trends in selection in those three country groups AT/CZ, CH, and UK (Fig. 4), hardly any development of PH_max_, *r*_max_, and $t_{PH_{stop}}$ was visible for genotypes registered in UK and AT/CZ. In contrast, the selection activity in CH resulted in strong and independent changes of lrc and $t_{PH_{stop}}$, and closely related changes of PH_max_ and *r*_max_. Selection in AT/CZ affected lrc without affecting other traits. Different trends emerged when analyzing the traits $t_{PH_{start}}$ and *T*_min_. Here, CH and AT/CZ genotypes hardly showed any development over time, but selection activities in UK have shifted *T*_min_ to lower values, also altering $t_{PH_{start}}$ to a later timing of jointing.

### Trait correlations confirm connection between temperature response, phenology, and height

Based on genetic correlations (Fig. 4), it became evident that PH_max_ is driven by temperature response parameters (*T*_min_, *r*_max_, and lrc) and a delayed end of stem elongation ($t_{PH_{stop}}$), indicated by moderate to strong correlations. For the correlations between the temperature response parameters themselves, *r*_max_ was strongly correlated to *T*_min_ and lrc. Nevertheless, there was only a moderate correlation between *T*_min_ and lrc, indicating that they can be selected partly independently. The end of stem elongation was moderately to strongly correlated with all three temperature response parameters and $t_{PH_{start}}$.

In summary, a stronger growth at the temperature optimum and a flat response to temperature delayed the end of stem elongation, which led to taller plants. A lower minimum temperature of growth delayed the end of stem elongation as well, but resulted in smaller plants. In any case, delaying the end of stem elongation has also delayed jointing.

So far, the reported correlations were based on genetic correlation calculations. Phenotypic correlations for individual years generally showed the same direction but differed in strength, with genetic correlations often being stronger (Fig. 4). This finding indicates confounding year effects that can be compensated for when screening multiple environments. Confounding effects were very evident for the correlations between the temperature response parameters and $t_{PH_{stop}}$ for which phenotypic correlations were weak but genetic correlations strong to very strong. In two situations, phenotypic and genetic correlations were contradictory (*r*_max_ versus *T*_min_, lrc versus *T*_min_) with moderate to strong genetic correlations. For two other situations, phenotypic and genetic correlations were contradictory (*r*_max_ versus $t_{PH_{start}}$, PH_max_ versus $t_{PH_{start}}$) but genetic correlations were only weak anyway.

### Genome-wide association studies reveal stable markers for across-year adjusted genotype means

The number of detected MTAs varied greatly among traits, year–sites, and depending on the applied model (Supplementary Figs S4–S11). No significant MTAs were detected for *T*_min_ for 2015, 2016, and the across-year BLUEs (Supplementary Fig. S4). Note that for $t_{PH_{start}}$ the trait values (BLUEs) for 2015 and 2016 were missing, thus preventing the calculation of MTAs. For all traits except $t_{PH_{stop}}$ and PH_max_, the highest numbers of MTAs were detected using the across-year BLUPs. Among the three applied models, the highest numbers of significant MTAs were detected using Blink, followed by FarmCPU. The MLM method only detected one significant MTA for the trait PH_max_ in 2017. There was considerable overlap in the detected MTAs between the three GWAS models within single year–sites and traits, as indicated by the sum of unique MTAs detected in one or more GWAS models (Supplementary Fig. S4; Supplementary Table S1). Apart from the overlap, there was also a considerable amount of MTA detected exclusively using FarmCPU or Blink, respectively. Yet, based on the inspection of quantile–quantile plots, FarmCPU and Blink appeared equally adequate in the control of false positives and false negatives (Supplementary Figs S4–S11).

Considering significant MTAs regardless of the GWAS model, stable markers consistently associated across several of the six analyzed models were investigated (four single year–site models for BLUE, one across-year model for BLUE, one across-year model for BLUP) for each trait. Most stable markers were detected for PH_max_, where 11 of the 23 unique MTAs in total were detected across 2–6 analyzed models (Supplementary Figs S5–S11; Supplementary Table S2). For the phenology traits $t_{PH_{start}}$ and $t_{PH_{stop}}$, four and eight stable MTAs were detected, respectively. Among the temperature response traits, four stable MTAs were found for *r*_max_ and two for lrc, whereas no stable MTAs were detected for *T*_min_. The linear model for temperature response *lm*_slope_ yielded five stable MTAs. With the multivariate GWAS, considerably fewer significant MTAs were detected compared with the univariate GWAS, and significant MTAs were only detected in the 2017 and 2018 BLUEs and across-year BLUPs (Supplementary Fig. S12). The multivariate GWAS on the temperature response traits together with PH_max_ (SupplementaryFig. S12, top) yielded four significant MTAs. The multivariate GWAS on the temperature response traits together with PH_max_ (Supplementary Fig. S12, bottom) yielded one significant MTA.

### Common marker–trait associations between different traits reflect genetic correlations

**Fig. 5.**
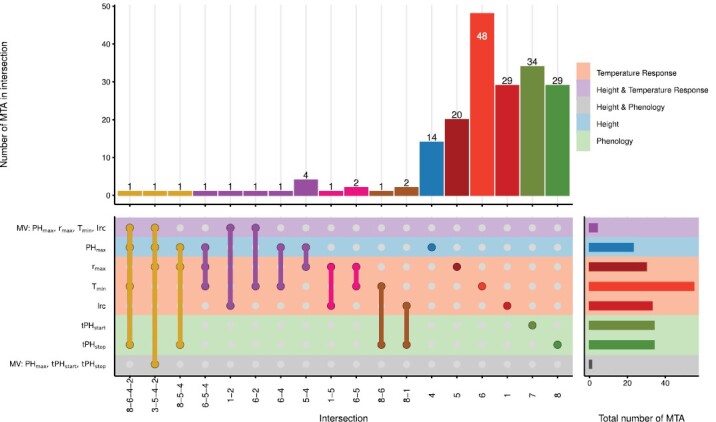
UpSet plot (Lex *et al.*, 2014; Krassowski *et al.*, 2021) depicting overlapping marker–trait associations (MTAs) between the GWAS for the temperature response traits *r*_max_, *T*_min_, and lrc, the phenology traits $t_{PH_{start}}$ and $t_{PH_{stop}}$, PH_max_, and two multivariate GWAS combining temperature response parameters with PH_max_, and combining PH_max_ with $t_{PH_{start}}$ and $t_{PH_{stop}}$.

To see whether the genetic correlations found in the phenotypic data are reflected in the GWAS results, all MTAs—irrespective of the year the association was detected and the GWAS model—were compared among traits. Most detected associations were unique to the respective trait. However, 17 markers in total were significantly associated in multiple univariate and multivariate GWAS models (Fig. 5).

The strong genetic correlation between *r*_max_ and PH_max_ was confirmed by four common MTAs (Fig. 5, intersection 5–4). Nevertheless, one common MTA for *T*_min_ and PH_max_ (intersection 6–4) and one common MTA for *T*_min_, *r*_max_, and PH_max_ (intersection 6–5–4) indicated that growth at optimum temperature was not the only driver of final height. Indeed, one common MTA each was found between the multivariate GWAS for temperature response traits/PH_max_ and the two narrow-sense temperature response parameters *T*_min_ and lrc, respectively (intersections 1–2 and 6–2), but no common MTA was found for *r*_max_. Although lrc and *T*_min_ were moderately genetically correlated, they shared no common MTA. Nevertheless, their genetic correlations to *r*_max_ were confirmed by one and two common MTAs, respectively, with *r*_max_ (intersections 1–5 and 6–5).

The significant genetic correlations of *T*_min_ and lrc to the phenology trait $t_{PH_{stop}}$ were confirmed by one and two common MTAs, respectively (intersections 8–6 and 8–1). The connection of $t_{PH_{stop}}$ to the temperature response parameters but also PH_max_ was further confirmed by one common MTA among the multivariate GWAS for temperature response traits/PH_max_, *T*_min_, PH_max_, and $t_{PH_{stop}}$ (intersection 8–6–4–2) as well as one common MTA among the multivariate GWAS for phenology traits/PH_max_, *r*_max_, PH_max_, and the multivariate GWAS for temperature response traits/PH_max_ (intersection 3–5–4–2). The common MTA between *r*_max_, PH_max_, and $t_{PH_{stop}}$ (intersection 8–5–4) indicated that growth at optimum temperature may also influence phenology traits, independent of narrow-sense temperature response parameters.

Together, these results confirm that the investigated traits are largely independent of a genomic level. Nevertheless, there are common factors between height and temperature response, temperature response and phenology, as well as factors shared among all three trait groups, reflecting the pattern found in the correlation analysis.

**Fig. 6.**
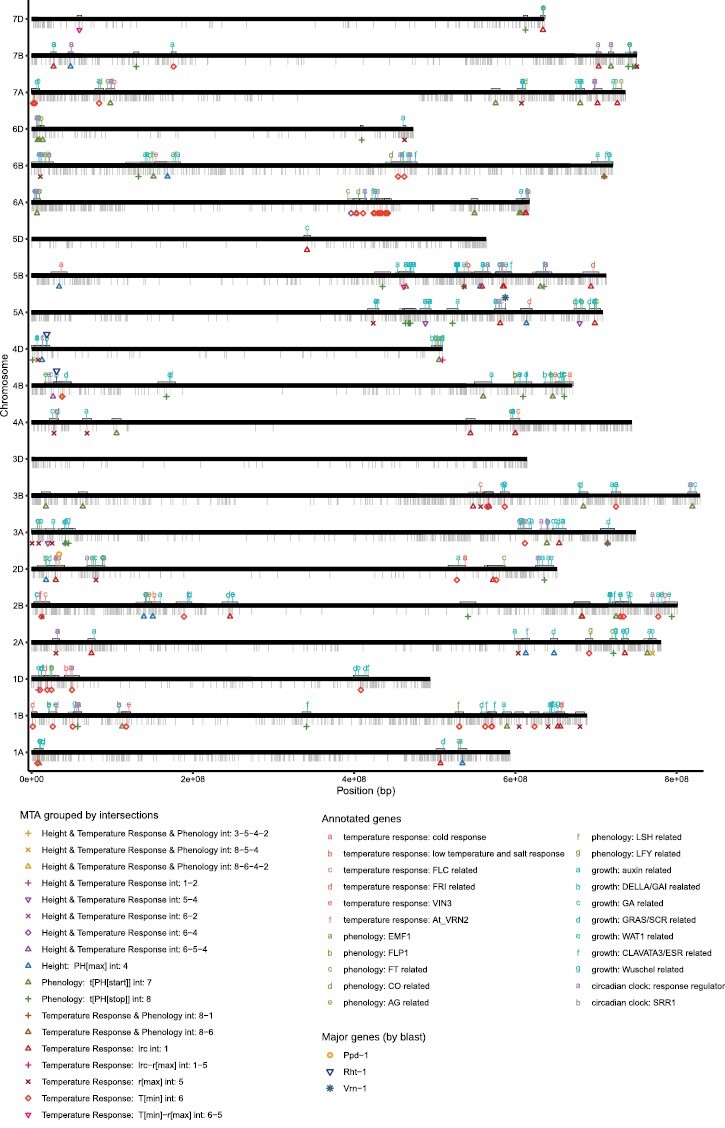
Chromosome plot depicting all SNP markers (gray ticks, lower side of chromosomes), detected MTAs grouped by trait category intersections (lower side of chromosomes; see color key), and potentially interesting gene motifs (upper side of chromosomes; see color key) that were found in the IWGSC refseqv1.0 functional annotation within chromosome-specific LD windows around the respective MTA (indicated by gray boxes on the upper side of the chromosomes).

Analyzing and discussing underlying genes for the detected MTA in detail is beyond the scope of this work. Nevertheless, we searched the IWGSC refseq1.0 (Appels *et al.*, 2018) functional annotation within chromosome-specific LD windows around each MTA (on average 7 Mb, Supplementary Dataset S1; an overview is given in Fig. 6). To briefly name the most prominent genes: we detected Rht-B1 in the vicinity of Tdurum_contig64772_417 (*T*_min_–*r*_max_–PH_max_ intersection 6–5–4, distance 4.3 Mb), Tdurum_contig33737_157 (*T*_min_ MTA, distance 6.8 Mb), and RAC875_rep_c105718_672 [*r*_max_–PH_max_–MV(i)–MV(ii) intersection 3–5–4–2, distance 7.4 Mb]. Rht-D1 was found 6 Mb upstream of PH_max_ MTA Kukri_rep_c68594_530, and Ppd-D1 was found 4.1 Mb upstream of lrc MTA Excalibur_c20196_503. Furthermore, we detected Vrn-A1 6.3 MB upstream of the lrc quantitative trait locus (QTL) wsnp_Ra_c12183_19587379. These genes were mapped to the IWGSC refseq1.0 using blastn. Around the remaining MTAs, we detected an increased presence of genes associated with growth [i.e. gene motifs related to auxin and gibberellin (DELLA/GAI) signal transduction pathways, as well as motifs related to GRAS/SCARECROW, WALLS ARE THIN 1, CLAVATA3/ESR and WUSCHEL]; phenology [i.e. gene motifs related to FLOWERING LOCUS T (FT), CONSTANS (CO), AGAMOUS (AG), EMBRYONIC FLOWER 1 (EMF1), Flowering-promoting factor 1-like protein 1 (FLP1), LIGHT-DEPENDENT SHORT HYPOCOTYLS (LSH), and LAFY (LFY)]; temperature response [i.e. FLOWERING LOCUS C (FLC), FRIGIDA (FRI),VERNALIZATION INSENSITIVE 3 (VIN3), VERNALIZATION 2 (At_VRN2), cold response- and low temperature- and salt response-associated proteins]; and motifs associated with the circadian clock [i.e. response regulators and SENSITIVITY TO RED LIGHT REDUCED 1 (SRR1)]. However, no clear pattern emerged as to the trait groups of the respective MTA and the gene motifs found nearby (Fig. 6).

## Discussion

### Field-based phenotyping allows extraction of robust basic physiological traits

In a previous study, it was shown that frequent and accurate canopy height measurements enable the extraction of phenological stages as well as temperature response parameters (Kronenberg *et al.*, 2020a). The latter were extracted using linear regressions between growth rates and average temperatures in the measurement interval. Within the observed data, the extracted temperature response parameters were highly heritable and allowed an accurate prediction of final height (Kronenberg *et al.*, 2020a). However, as the model did not account for the non-linearity of the temperature response, the interpretability of the parameters was limited. In addition, Kronenberg *et al.* (2020a) used averaged temperatures, disregarding temperature fluctuations during measurement intervals. Considering the diurnal temperature pattern is of particular significance: height measurements are usually done every few days and hence the measured growth between time points is the result of multiple diurnal covariate cycles, such as temperature courses. Aggregating these temperature courses to the frequency of canopy height measurements shrinks the observed temperature distribution towards the mean (Roth *et al.*, 2022b). In soybean, diurnal temperature patterns have been shown to strongly affect leaf growth as well as carbohydrate metabolism and gene expression (Kronenberg *et al.*, 2020b). Based on simulations, Roth *et al.* (2022b) demonstrated that using an asymptotic dose–response model and optimizing the parameters based on temperature courses instead of mean temperatures allows a more accurate description of temperature response in the stem elongation phase of winter wheat.

The results of this study demonstrate the applicability of such an asymptotic model for field-derived data. Both fixed platform and UAS-based canopy height measurements were equally suited for this purpose. While repeatabilities varied depending on the year, across-year heritabilities were high. Apparently, a skewed distribution of temperatures towards very high temperatures decreased the repeatability of *r*_max_, a skewed distribution towards low temperatures decreased the repeatability of *T*_min_, and frequent temperatures in the mid-range decreased the repeatability of lrc. These limitations may be seen as systematic artifacts of fitting a temperature response to measurements with irregular measurement frequencies and seasonal temperature courses (Roth *et al.*, 2022b). None of the seasons showed all these characteristics at the same time, but none was completely free of them either.

Consequently, depending on the temperature constellation in the respective environment, temperature response parameters are difficult to quantify with high precision. However, high across-year heritabilities and a high number of significant MTAs for across-year BLUPs indicated that genotype-by-year interactions are small compared with genotype effects. Hence, temperature dose–response parameters represent robust basic physiological traits if monitored in multi-year trials. Roth *et al.* (2023) could show that such basic physiological traits are correlated to manually measured phenology parameters, and allow the phenomic prediction of yield, yield stability, and protein content for a Swiss elite winter wheat set. The reported heritabilities and genetic correlations of this study of phenology and temperature response traits are in accordance with those reported in Roth *et al.* (2023), raising hope that similar methods will also work on less diverse genotype sets.

### Temperature response traits are independent drivers of phenology and height

The results revealed a clear region-driven structure within the observed population regarding the origin of the genotypes. The findings indicate that final height, phenology, and temperature response traits are related to the adaptation to various climatic regions and production systems. A connection among temperature response, phenology, and height was previously reported (Kronenberg *et al.*, 2020a). While the current results confirmed these findings, using the asymptotic temperature response model further allowed dissection and clarification of temperature response relationships and their genetic make-up.

The genetic correlations and concurring shared MTAs among *r*_max_, PH_max_, $t_{PH_{start}}$, and $t_{PH_{stop}}$ indicated some common genetic basis. The GWAS results further confirmed that all of these traits are highly quantitative and only a fraction of the detected QTLs are shared among these traits. With respect to the applied GWAS models, our results show that multi-locus models appear more adequate in terms of power compared with MLM to detect associations with complex traits in wheat. This is in accordance with results from soybean and maize (Kaler and Purcell, 2019).

It is known that the stem elongation rate of genotypes with comparable phenology but different final heights (e.g. near-isogenic lines for gibberellin-insensitive dwarfing genes) differs (Youssefian *et al.*, 1992). The effect of reduced height genes on growth rates was confirmed by the strong genetic correlation between *r*_max_ and PH_max_, but the correlations with other parameters and common shared MTAs indicated that dwarfing genes were not the only driver of the growth rate.

In their study investigating the effect of breeding on temperature response, Parent and Tardieu (2012) normalized the elongation rates at optimal temperatures (or better to say at 20 °C which is close to the optimum). This was done to enable the comparison of different growth processes at different scales. However, the absolute growth at optimal temperature is a relevant component of plant adaptation. In contrast to the functional temperature response model used by Parent and Tardieu (2012), the asymptotic temperature response model used in this study allowed insights to be gained on the base temperature of growth, the steepness of the response, and the maximum growth rate *r*_max_.

The parameter *r*_max_ is not a temperature response in the narrow sense but rather represents the growth at the temperature optimum. In contrast, the curvature parameter lrc and the base temperature *T*_min_ may indicate temperature dependencies of growth. It is not within the scope of this study to explore and elucidate the physiological basis of these traits. Nevertheless, a contextualization is given here for the interested reader. Although there has been significant progress regarding the molecular mechanisms of temperature sensing and their integration in signal transduction and response pathways in Arabidopsis, the understanding of temperature response remains limited (Wigge, 2013; Vu *et al.*, 2019; Kerbler and Wigge, 2023). In Arabidopsis, the transcription factor PHYTOCHROME INTERACTING FACTOR 4 (PIF4) has been identified as a central hub integrating temperature and light signals and controlling transcriptional and post-transcriptonal regulation of plant thermomorphogenesis (Quint *et al.*, 2016; Jin *et al.*, 2020). PIF4 is regulated by the circadian clock genes ELF4, ELF3, LUX, and TOC1 (Nusinow *et al.*, 2011; Zhu *et al.*, 2016; Gangappa and Kumar, 2017), and acts on a large number of genes related to phytohormone biosynthesis and growth (Quint *et al.*, 2016; Martínez *et al.*, 2018). Furthermore, PIF4 interacts with the flowering pathway involving the genes FLC, FRI, FT, and CO (Proveniers and van Zanten, 2013; Fernández *et al.*, 2016; Jenkitkonchai *et al.*, 2021). In contrast, very little is known about the physiological basis of ambient temperature response in wheat. The protein kinase MAP4K4/TOT3 has been found to be required for thermomorphogenesis in wheat (Vu *et al.*, 2021). Furthermore an interaction effect between ELF3 expression and temperature on heading date has been reported in wheat (Ochagavía *et al.*, 2019). Dixon *et al.* (2019) reported that VRN2 and ODDSOC2 are re-activated after vernalization when wheat experiences warmer temperature, and Kiss *et al.* (2017) showed that the expression of VRN1, VRN2, and PPD1 was affected by ambient temperature and photoperiod, and correlated with plant development.

In the current study, we found the major genes VRN1, RHT1, and PPD1, as well as several predicted gene motifs related to growth, phenology, temperature response, and the circadian clock in the vicinity of SNPs associated with different temperature response and phenology traits as well as final height. However, the experimental set-up and the applied methods do not allow us to draw any conclusions regarding any association between these gene motifs and the investigated traits, as illustrated in Fig. 6. The size of LD (on average 7 Mb) means that a large number of gene motifs are potentially associated with a specific MTA. Furthermore, the clustering of MTAs from different traits in specific chromosomal regions causes the LD windows to overlap, making it impossible to disentangle individual MTAs and underlying genomic regions. The same applies to putative pleiotropic structures with respect to shared MTAs between individual traits and multivariate GWAS. Even though the genetic correlations in conjunction with shared univariate and multivariate MTAs may point towards pleiotropy, the size of LD does not allow any such conclusion to be drawn.

Thus, further research is warranted to elucidate the underlying physiological mechanisms of these traits. In order to do so, the use of a designed bi-parental population in combination with QTL mapping and gene expression assays may be applied in future studies. In the meantime, phenomic approaches in combination with genomic selection may provide the means to further investigate temperature response traits under field conditions.

Based on genetic correlations and shared MTAs, one can conclude that increased *T*_min_ leads to an early end of stem elongation while only marginally affecting PH_max_. Importantly, lrc has a strong connection to *r*_max_, but a less strong connection to PH_max_ and no common MTAs, while *r*_max_ has a strong correlation to PH_max_ and shares four MTAs. Therefore, adapting lrc (and, according to genetic correlations, also *T*_min_) seems to have little side effect on final height. Interestingly, the steepness of response lrc and *T*_min_ have a stronger correlation to the end of stem elongation than *r*_max_. Consequently, temperature response in the narrow sense (*T*_min_ and lrc) is more closely connected to phenology than growth at optimum temperatures.

As these narrow-sense temperature response parameters appear to be partly genetically independent, these traits may be of key interest for breeding. Both final height and phenology are key traits of local adaptation. Selection for specific temperature response trait combinations may thus allow phenology and height to be independently adjusted, offering opportunities towards improved adaptation to specific environments. On the downside, low heritabilities and genomic prediction accuracies for *T*_min_ and lrc based on GBLUPs indicated potential difficulties in the selection process.

In the examined set of genotypes, both phenology and temperature response appear to drive local adaptation. For varieties registered in Great Britain, selection in breeding throughout the years 1970–2018 led to later jointing and to decreasing the minimum temperature of growth. These two features compensated each other with respect to generating varieties of comparable height that were registered throughout the years. For varieties registered in Switzerland, plant height decreased throughout the years, coinciding with an earlier end of stem elongation and a decrease of growth at optimum temperature. In Austria and the Czech Republic, final height, jointing, and end of stem elongation remained similar throughout the decades, but the steepness of temperature response increased. It has to be noted that the observed region-specific difference may be driven not only by climatic conditions (increase in temperature and overall decrease of water availability) but also by management and policy in the particular country. Yet, the data show that country-specific strategies for the development of phenology have been selected for, allowing yield potential to be maintained throughout the decades under the effect of a globally changing climate. Future studies need to reveal the physiological advantages that the observed, country-specific selection strategies have brought with them.

Yet, most clearly for the case example of varieties registered in Great Britain, the advantages of such selection strategies can be made clear: given the applicability of the concept of temperature sum, a warming climate leads to the possibility of accumulating the same biomass in a shorter period of time. Hence, the shift of jointing towards a later time of the year (within the limits of ensuring unstressed flowering) can be compensated. Moreover, the decreasing base temperature increases chances to grow demonstrably even during days with relatively low temperature that still remain frequent also towards later times of the season (Supplementary Fig. S3D).

## Conclusion

In this study, we could demonstrate that temperature response parameters are heritable traits with a strong physiological basis. HTFP allows the extraction of such response curves and timing parameters for jointing and the end of stem elongation. Flexible and affordable drone and RGB hardware is as suitable as stationary phenotyping platforms such as the FIP, allowing breeders to scale up phenotyping to large breeding populations.

Nevertheless, response parameters are occasionally difficult to quantify with high precision in the field, as the efficiency of HTFP will depend on temperature fluctuation during stem elongation. Combining multiple years will mitigate these limitations.

Analyzing the dependencies of traits and population structures revealed that breeding indeed has affected the phenology and temperature response of the stem elongation phase of wheat. Genotypic variances in both (narrow-sense) response to temperature and growth rates at the optimum were indicated. Final height was driven not only by the maximum growth rate at the optimum, but also by phenology and by the responsiveness to temperature between cardinal temperatures. A high number of MTAs were detected for temperature response traits, highlighting their quantitative nature. Although not equally strong for all traits, the measured prediction accuracies promise a high potential of genomic selection approaches for temperature response and phenology traits.

## Supplementary data

The following supplementary data are available at *JXB* online.

Fig. S1. Experimental fields at the FIP site.

Fig. S2. Examples of Q–Q plots showing inflation and deflation, respectively, for the Blink and FarmCPU models with three principal components (3PC) and omitted principal components (0PCs).

Fig. S3. Fitted curves to height data.

Fig. S4. Number of detected significant marker–trait associations (MTAs) among the three univariate GWAS models.

Fig. S5. Manhattan plots and quantile–quantile plots depicting the GWAS results for *r*_max_.

Fig. S6. Manhattan plots and quantile–quantile plots depicting the GWAS results for *T*_min_.

Fig. S7. Manhattan plots and quantile–quantile plots depicting the GWAS results for lrc.

Fig. S8. Manhattan plots and quantile–quantile plots depicting the GWAS results for *lm*_slope_.

Fig. S9. Manhattan plots and quantile–quantile plots depicting the GWAS results for PH_max_.

Fig. S10. Manhattan plots and quantile–quantile plots depicting the GWAS results for $t_{PH_{stop}}$.

Fig. S11. Manhattan plots and quantile–quantile plots depicting the GWAS results for $t_{PH_{start}}$.

Fig. S12. Manhattan plots and quantile–quantile plots depicting the multivariate GWAS results.

Table S1. Total number of significant marker–trait associations (MTAs) for each trait across all years and for all traits in each year.

Table S2. Stable marker–trait associations (MTAs) over years.

Dataset S1. IWGSC refseq1.0 functional annotation within chromosome-specific LD windows around each MTA.

erad481_suppl_Supplementary_Figures_S1-S12_Tables_S1-S2

erad481_suppl_Supplementary_Dataset_S1

## Data Availability

Unprocessed data are available from the authors upon reasonable request. Source codes that support the findings of this study are openly available in the ETH gitlab repository at https://gitlab.ethz.ch/crop_phenotyping/htfp_data_processing.
