# Supplementary material for: High-throughput field phenotyping reveals that selection in breeding has affected the phenology and temperature response of wheat in the stem elongation phase
Source: J Exp Bot. 2023 Dec 22;75(7):2084–99. doi: 10.1093/jxb/erad481 (PMC10967243; doi:10.1093/jxb/erad481)
Supplement: erad481_suppl_Supplementary_Figures_S1-S12_Tables_S1-S2 [file erad481_suppl_supplementary_figures_s1-s12_tables_s1-s2.pdf]

Supplementary Figures and Tables

The following supplementary data are available in this document:

- Figure S1. Experimental fields at the FIP site.
- Figure S2. Examples of Q-Q plots showing inflation respectively deflation for the Blink and FarmCPU models with 3 principal components (3PC) and omitted principal components (0PC).
- Figure S3. Fitted curves to height data.
- Figure S4. Number of detected significant marker-trait associations (MTAs) among the three univariate GWAS models.
- Figure S5. Manhattan plots and quantile–quantile plots depicting the GWAS results for r_{max}.
- Figure S6. Manhattan plots and quantile–quantile plots depicting the GWAS results for T_{min}.
- Figure S7. Manhattan plots and quantile–quantile plots depicting the GWAS results for *lrc*.
- Figure S8. Manhattan plots and quantile–quantile plots depicting the GWAS results for lm_{slope}.
- Figure S9. Manhattan plots and quantile–quantile plots depicting the GWAS results for PH_{max}.
- Figure S10. Manhattan plots and quantile–quantile plots depicting the GWAS results for $t_{\text{PH}_{\text{stop}}}$.
- Figure S11. Manhattan plots and quantile–quantile plots depicting the GWAS results for $t_{\text{PH}_{\text{start}}}$.
- Figure S12. Manhattan plots and quantile–quantile plots depicting the multivariate GWAS results.
- Table S1. Total number of significant marker trait associations (MTAs) for each trait across all years and for all traits in each year.
- Table S2. Stable marker trait associations (MTAs) over years.

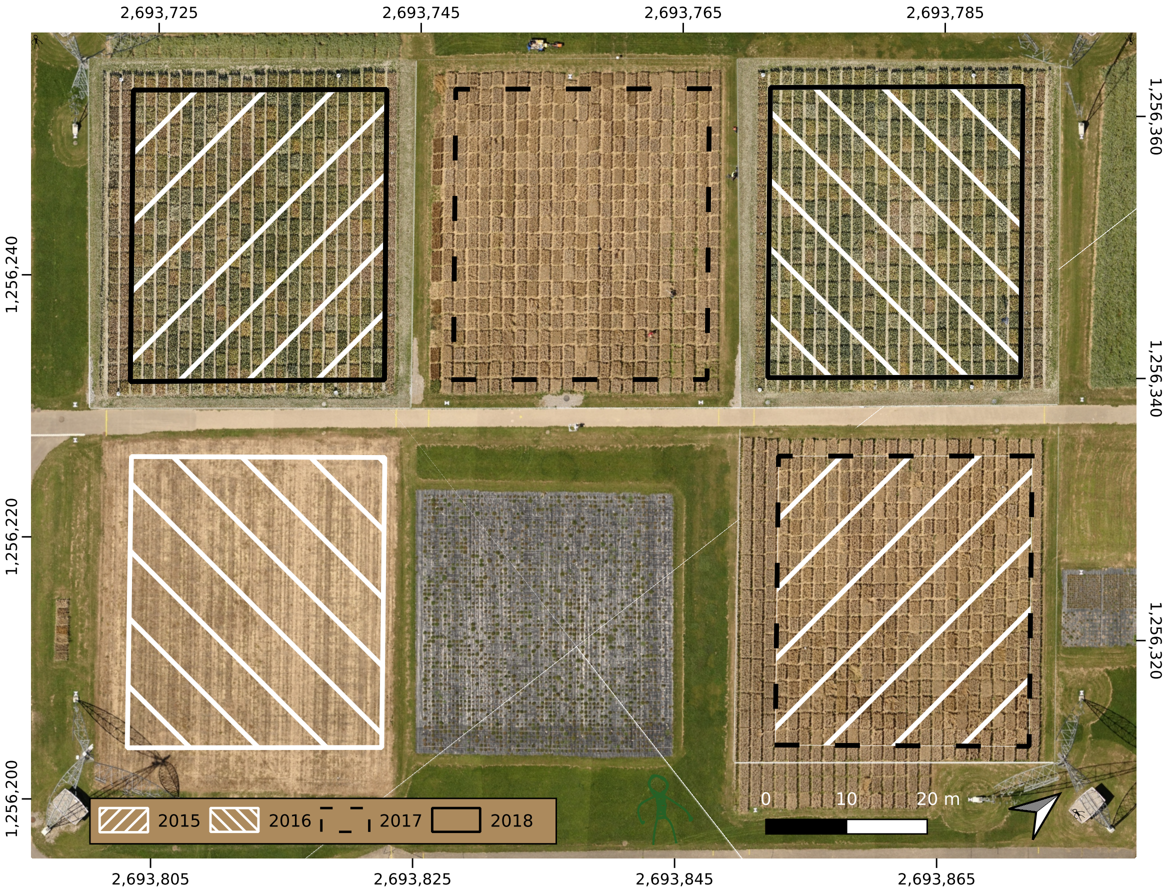

Source: Orthophoto 2017 (Background): Crop Science Group, ETH Zurich; Orthophoto 2018 (Top left/right): Trait Spotting Project
Coordinate reference system: EPSG:2056 - CH1903+ / LV95

Figure S1: Experimental fields at the FIP site with indications of the two replications per year (boxes) and aerial images of two years (2017, superimposed on 2018 top left and top right) as background.

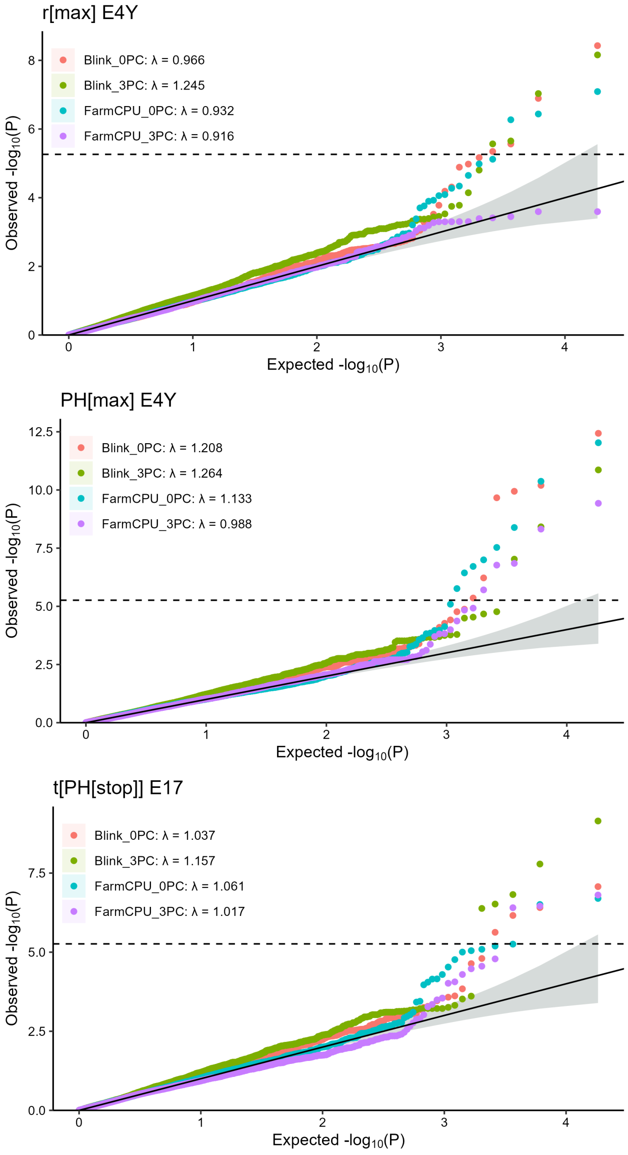

Figure S2: Examples of Q-Q plots showing inflation respectively deflation for the Blink and FarmCPU models with 3 principal components (3PC) and omitted principal components (0PC).

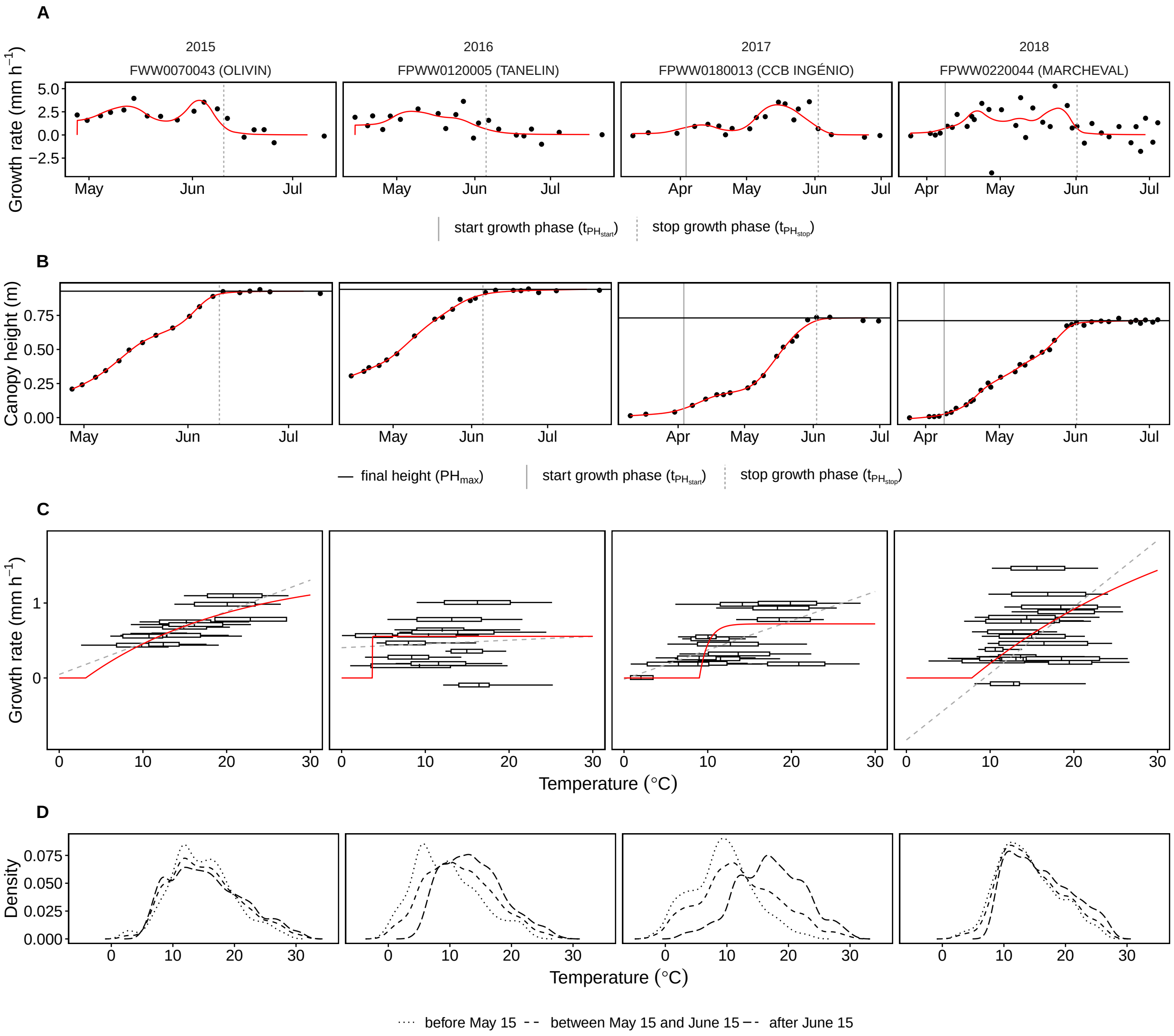

Figure S3: Growth rates (a), plant heights (b), fitted temperature dose-response curves (c), and corresponding distribution of measured temperatures (d) for four randomly selected genotypes in four growing seasons. The solid red lines indicate the fitted monotonous increasing P-spline (b), its first order derivative (a) and the fitted temperature dose-response (c), black solid points represent measurement values. In addition, the detected start and end of the growing phase (solid and dotted vertical lines) and the calculated final height (solid horizontal line) are indicated.

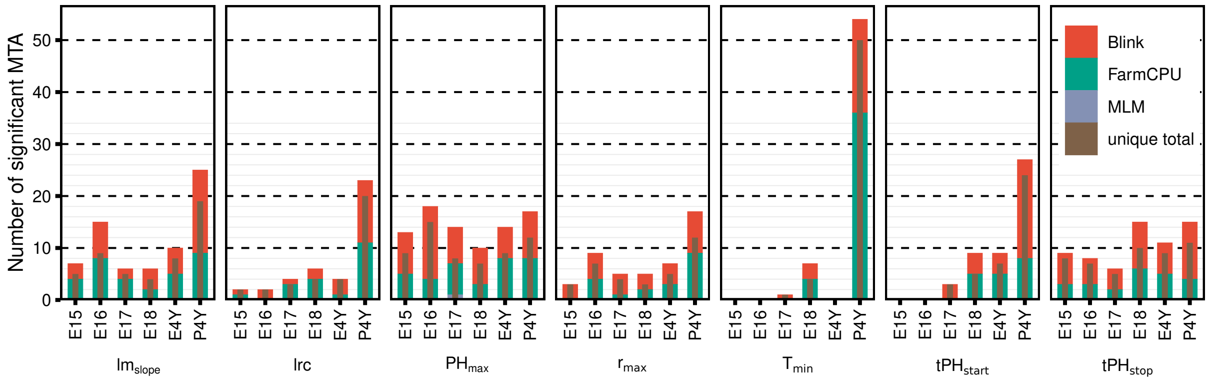

Figure S4: Number of detected significant marker-trait associations (MTAs) among the three univariate GWAS models (see color legend) and all traits and year genotype BLUEs (E15–E18) and across-year genotype BLUEs (E4Y) and BLUPs (P4Y). The brown bars indicate the total number of MTAs uniquely identifed within each trait-year combination among the three models.

Table S1: Total number of significant (LOD > 5.43) Marker trait associations (MTAs) for each trait across all years and for all traits in each year as well as the fraction detected by all three GWAS models (Blink, FarmCPU, MLM), two models or one model, respectively. For overlapping MTAs between the models, also non-significant MTAs (LOD > 3.00) were considered (Supplementary materials, Figures S5-S11)

trait	yearsite	number of singificant MTA	fraction detected in 3 models (%)	fraction detected in 2 models (%)	fraction detected in 1 model (%)
**all traits**	all yearsites	323	18	42	40
**lm_{slope}**	all yearsites	50	20	48	32
lrc	all yearsites	35	14	43	43
**PH_{max}**	all yearsites	60	27	47	27
**r_{max}**	all yearsites	34	29	47	24
**T_{min}**	all yearsites	55	7	20	73
**t_{PH}start**	all yearsites	39	8	33	59
**t_{PH}stop**	all yearsites	50	18	58	24
**all traits**	E15	27	30	33	37
**all traits**	E16	40	20	48	32
**all traits**	E17	29	24	48	28
**all traits**	E18	37	43	51	5
**all traits**	E4Y	42	26	57	17
**all traits**	P4Y	148	5	34	61

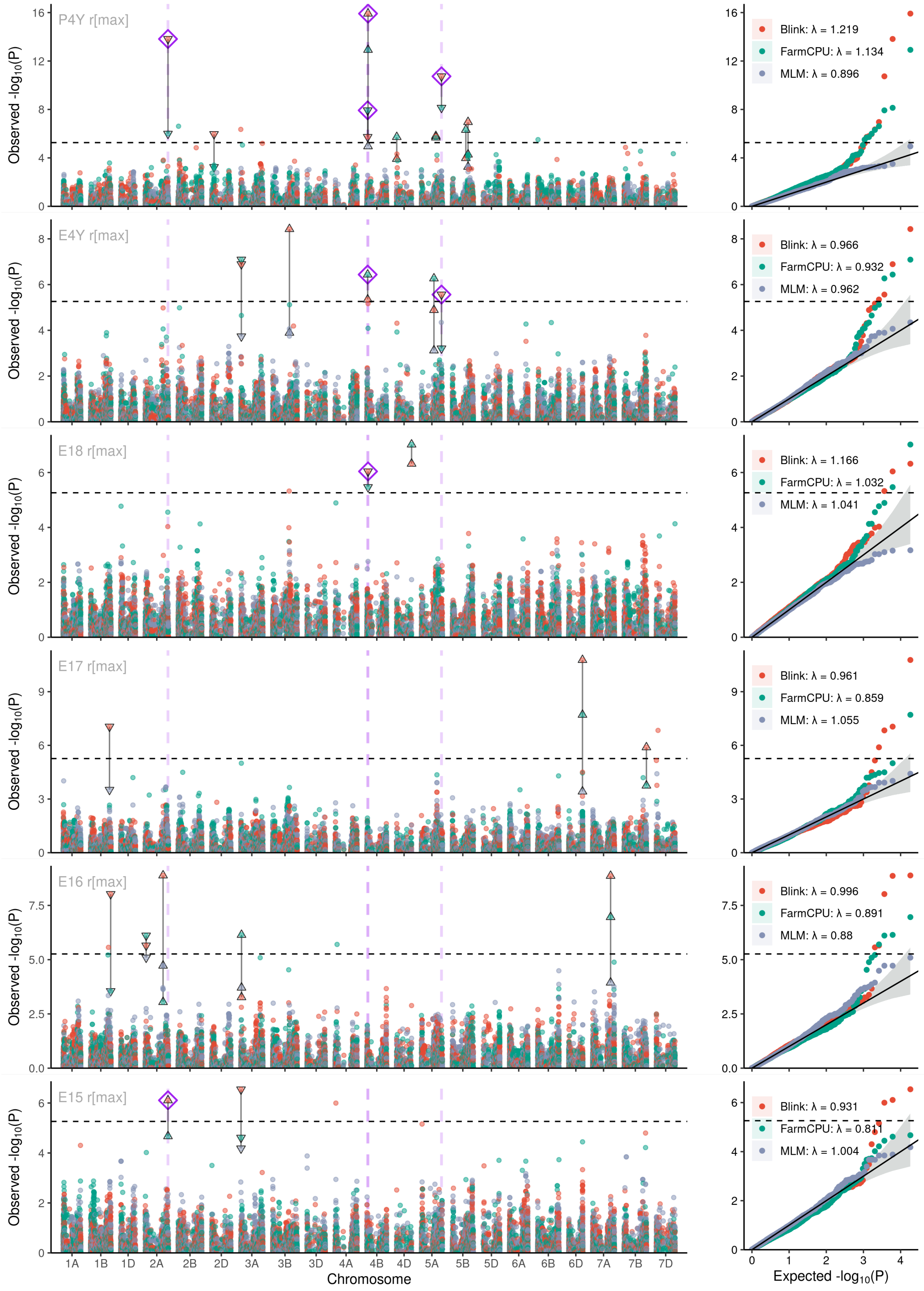

Figure S5: Manhattan plots and quantile–quantile plots depicting the GWAS results for $r_{\max}$ using the across-year BLUPs (a), across-year BLUES (b) or single-year BLUES in the respective year 2018 (c), 2017 (d), 2016 (e) and 2015 (f). In each plot, the results are given for all three applied models (Blink = red points, FarmCPU = green points, MLM = blue points) including the respective test statistic inflation factor (λ, see color legend). MTAs detected by multiple models within the respective year are indicated by black triangles connected with line segments. MTAs detected across multiple years irrespective of the GWAS model are indicated by purple rhombs and dashed vertical lines. The dashed horizontal lines indicate the Bonferroni corrected significance threshold for $\alpha = 0.05$.

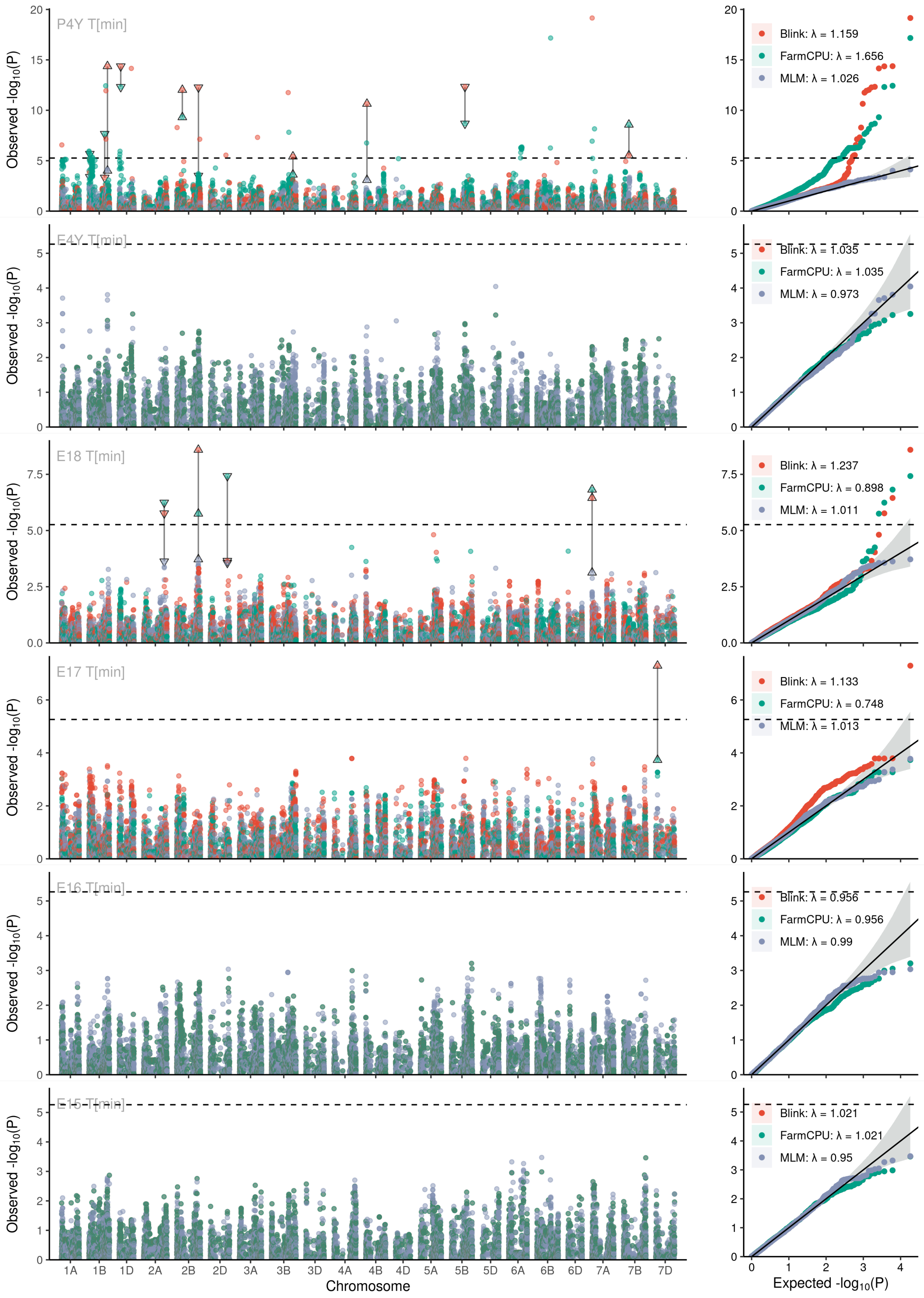

Figure S6: Manhattan plots and quantile–quantile plots depicting the GWAS results for $T_{\min}$ using the across-year BLUPs (a), across-year BLUES (b) or single-year BLUES in the respective year 2018 (c), 2017 (d), 2016 (e) and 2015 (f). In each plot, the results are given for all three applied models (Blink = red points, FarmCPU = green points, MLM = blue points) including the respective test statistic inflation factor (λ, see color legend). MTAs detected by multiple models within the respective year are indicated by black triangles connected with line segments. MTAs detected across multiple years irrespective of the GWAS model are indicated by purple rhombs and dashed vertical lines. The dashed horizontal lines indicate the Bonferroni corrected significance threshold for $\alpha = 0.05$. Note that the gwas resulst for Blink and FarmCPU are identical in case no significant pseudo-QTN are detected (Jiabo Wang, personal communication). Thus, Blink results in E4Y, E16 and E15 are not visible as they are overlaid by the FarmCPU results.

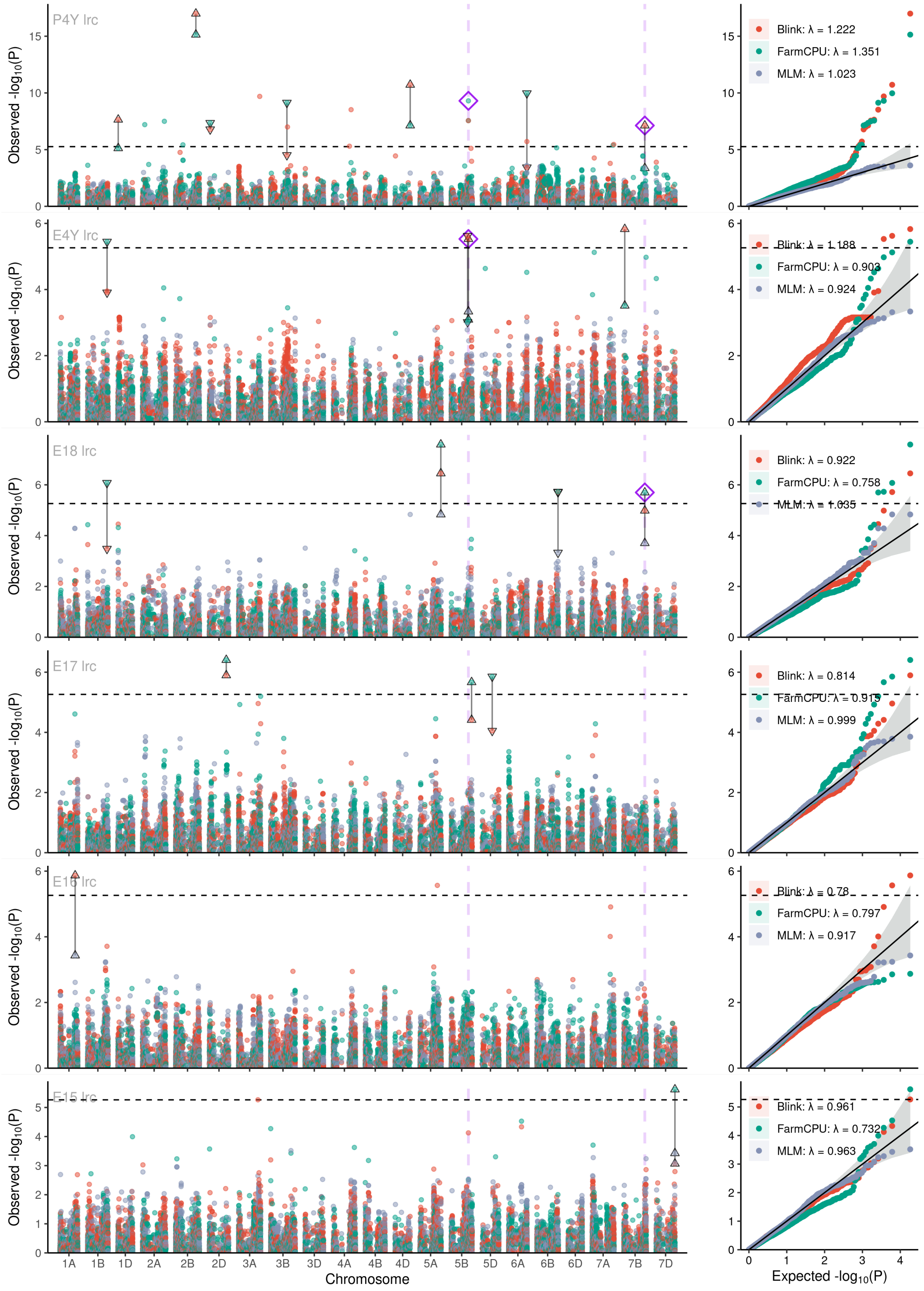

Figure S7: Manhattan plots and quantile–quantile plots depicting the GWAS results for *lrc* using the across-year BLUPs (a), across-year BLUES (b) or single-year BLUES in the respective year 2018 (c), 2017 (d), 2016 (e) and 2015 (f). In each plot, the results are given for all three applied models (Blink = red points, FarmCPU = green points, MLM = blue points) including the respective test statistic inflation factor (λ, see color legend). MTAs detected by multiple models within the respective year are indicated by black triangles connected with line segments. MTAs detected across multiple years irrespective of the GWAS model are indicated by purple rhombs and dashed vertical lines. The dashed horizontal lines indicate the Bonferroni corrected significance threshold for $\alpha = 0.05$. Note that the GWAS results for Blink and FarmCPU are identical in case no significant pseudo-QTN are detected (Jiabo Wang, personal communication)

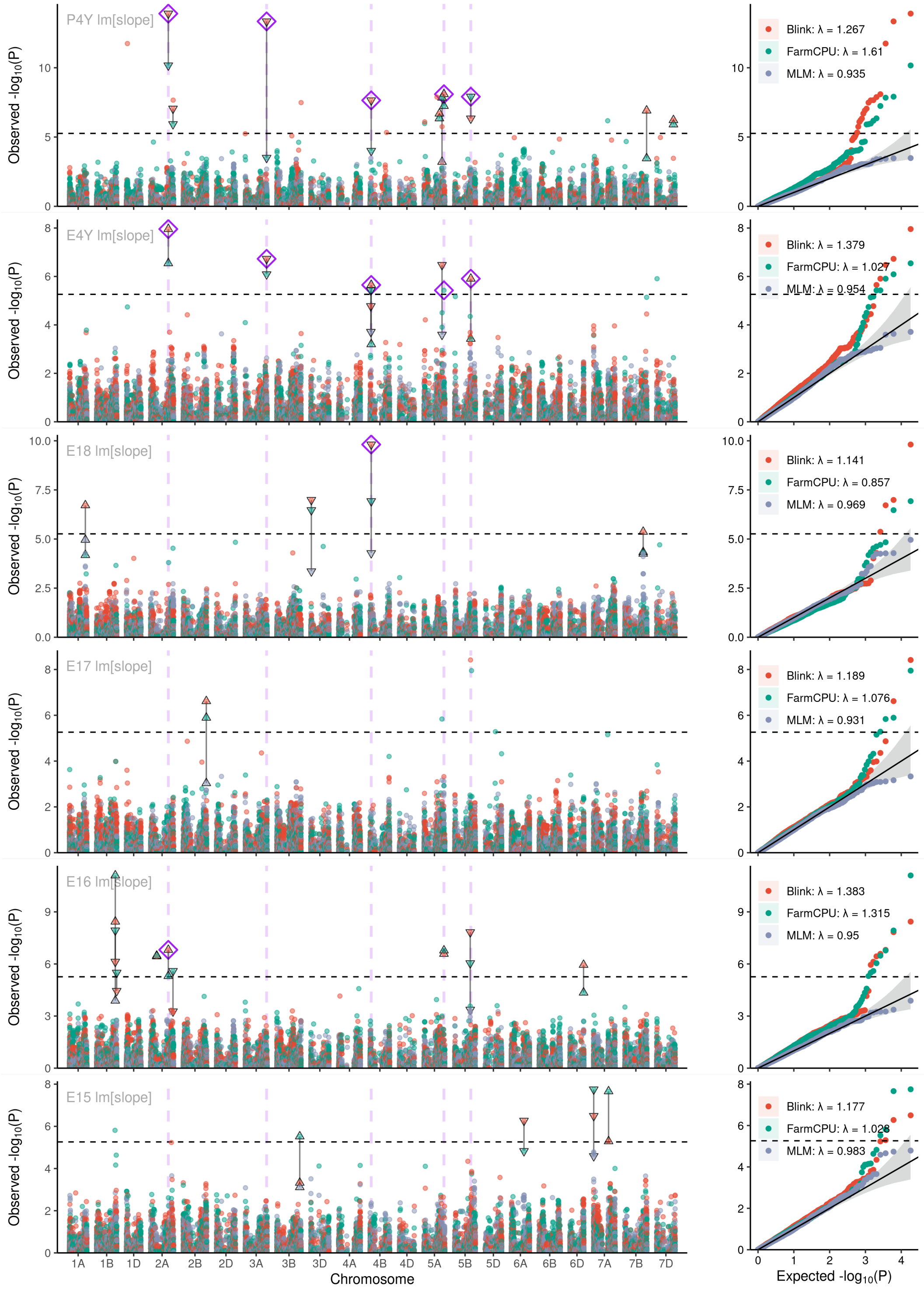

Figure S8: Manhattan plots and quantile–quantile plots depicting the GWAS results for linear temperature response model lm_{slope} using the across-year BLUPs (a), across-year BLUES (b) or single-year BLUES in the respective year 2018 (c), 2017 (d), 2016 (e) and 2015 (f). In each plot, the results are given for all three applied models (Blink = red points, FarmCPU = green points, MLM = blue points) including the respective test statistic inflation factor (λ, see color legend). MTAs detected by multiple models within the respective year are indicated by black triangles connected with line segments. MTAs detected across multiple years irrespective of the GWAS model are indicated by purple rhombs and dashed vertical lines. The dashed horizontal lines indicate the Bonferroni corrected significance threshold for $\alpha = 0.05$.

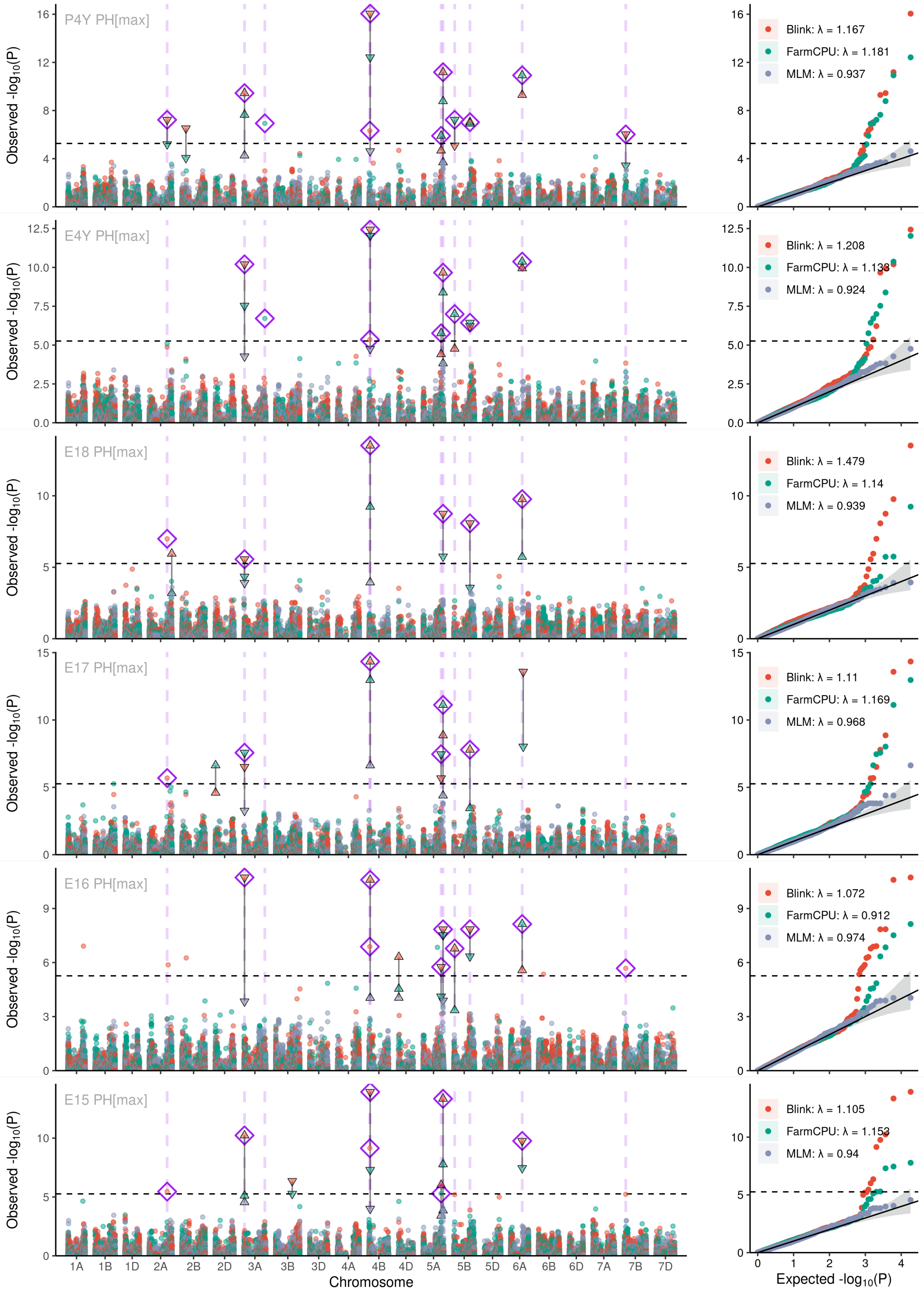

Figure S9: Manhattan plots and quantile–quantile plots depicting the GWAS results for $PH_{\max}$ using the across-year BLUPs (a), across-year BLUES (b) or single-year BLUES in the respective year 2018 (c), 2017 (d), 2016 (e) and 2015 (f). In each plot, the results are given for all three applied models (Blink = red points, FarmCPU = green points, MLM = blue points) including the respective test statistic inflation factor (λ, see color legend). MTAs detected by multiple models within the respective year are indicated by black triangles connected with line segments. MTAs detected across multiple years irrespective of the GWAS model are indicated by purple rhombs and dashed vertical lines. The dashed horizontal lines indicate the Bonferroni corrected significance threshold for $\alpha = 0.05$.

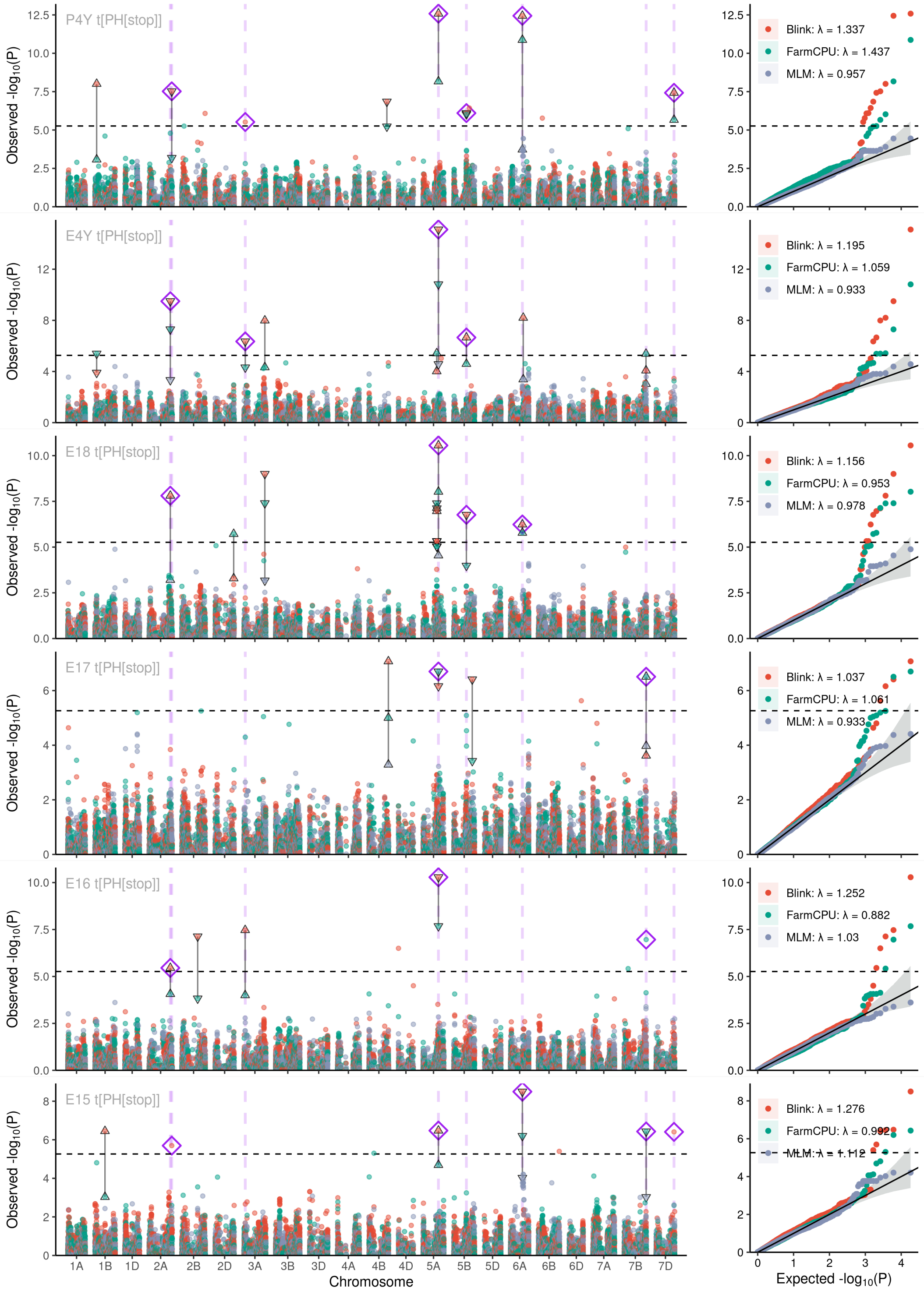

Figure S10: Manhattan plots and quantile–quantile plots depicting the GWAS results for $t_{\mathrm{PH}_{\mathrm{stop}}}$ using the across-year BLUPs (a), across-year BLUES (b) or single-year BLUES in the respective year 2018 (c), 2017 (d), 2016 (e) and 2015 (f). In each plot, the results are given for all three applied models (Blink = red points, FarmCPU = green points, MLM = blue points) including the respective test statistic inflation factor (λ, see color legend). MTAs detected by multiple models within the respective year are indicated by black triangles connected with line segments. MTAs detected across multiple years irrespective of the GWAS model are indicated by purple rhombs and dashed vertical lines. The dashed horizontal lines indicate the Bonferroni corrected significance threshold for $\alpha = 0.05$.

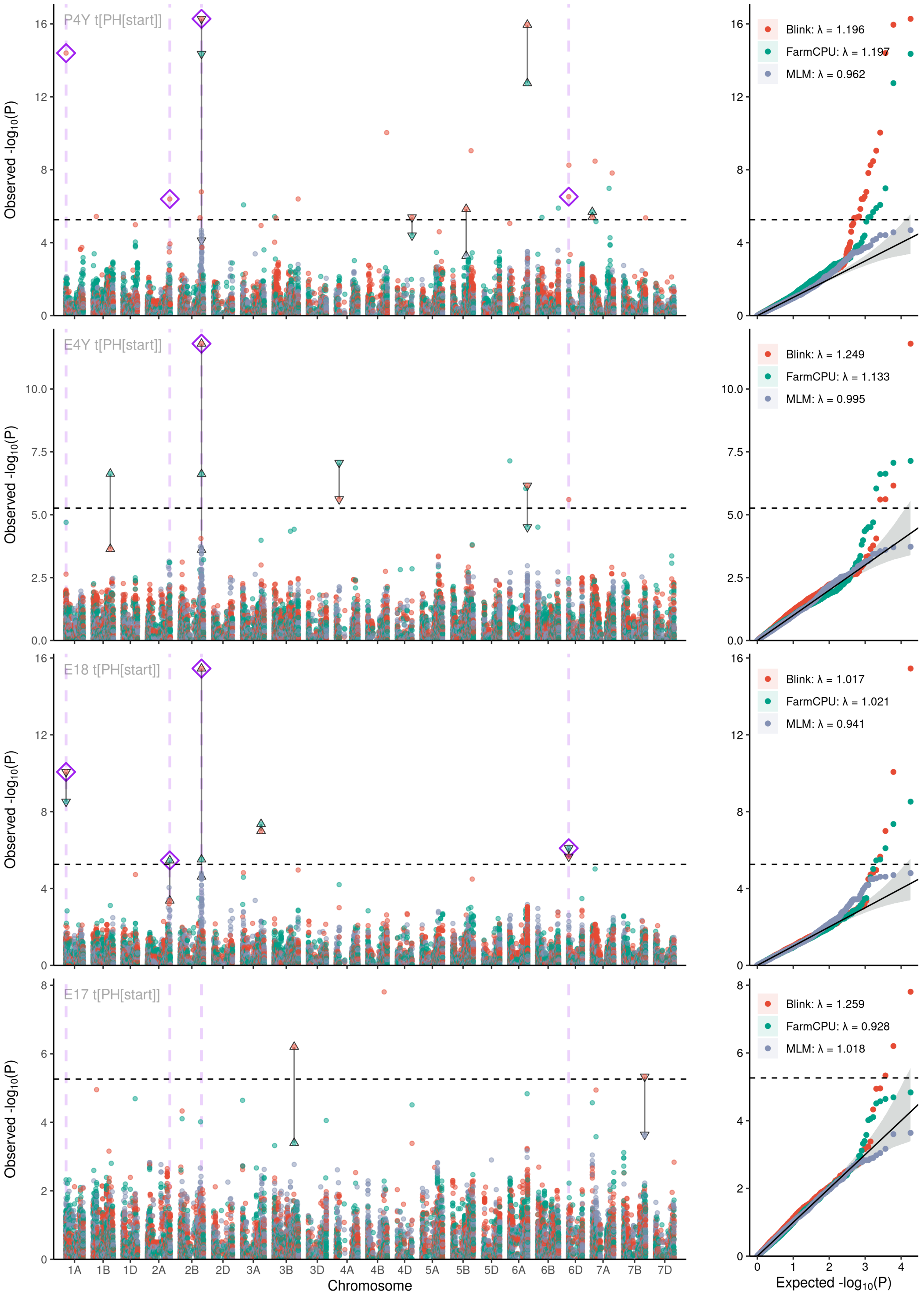

Figure S11: Manhattan plots and quantile–quantile plots depicting the GWAS results for $t_{\mathrm{PH}_{\mathrm{start}}}$ using the across-year BLUPs (a), across-year BLUES (b) or single-year BLUES in the respective year 2018 (c), 2017 (d), 2016 (e) and 2015 (f). In each plot, the results are given for all three applied models (Blink = red points, FarmCPU = green points, MLM = blue points) including the respective test statistic inflation factor (λ, see color legend). MTAs detected by multiple models within the respective year are indicated by black triangles connected with line segments. MTAs detected across multiple years irrespective of the GWAS model are indicated by purple rhombs and dashed vertical lines. The dashed horizontal lines indicate the Bonferroni corrected significance threshold for $\alpha = 0.05$.

Table S2: Number of marker trait associations detected across multiple years (including across-year BLUEs and BLUPs) for each trait.

trait	total number of MTA	total number of unique MTA	total number of multiple yearsite MTA	average number of yearsites per MTA	fraction of multiple yearsite MTA
**PH_{max}**	60	23	11	4	0.48
**$t_{PH}stop$**	50	34	8	3	0.24
**r_{max}**	34	30	4	2	0.13
**lm_{slope}**	50	43	5	2	0.12
**$t_{PH}start$**	39	34	4	2	0.12
lrc	35	33	2	2	0.06
**T_{min}**	55	55	0	1	0.00

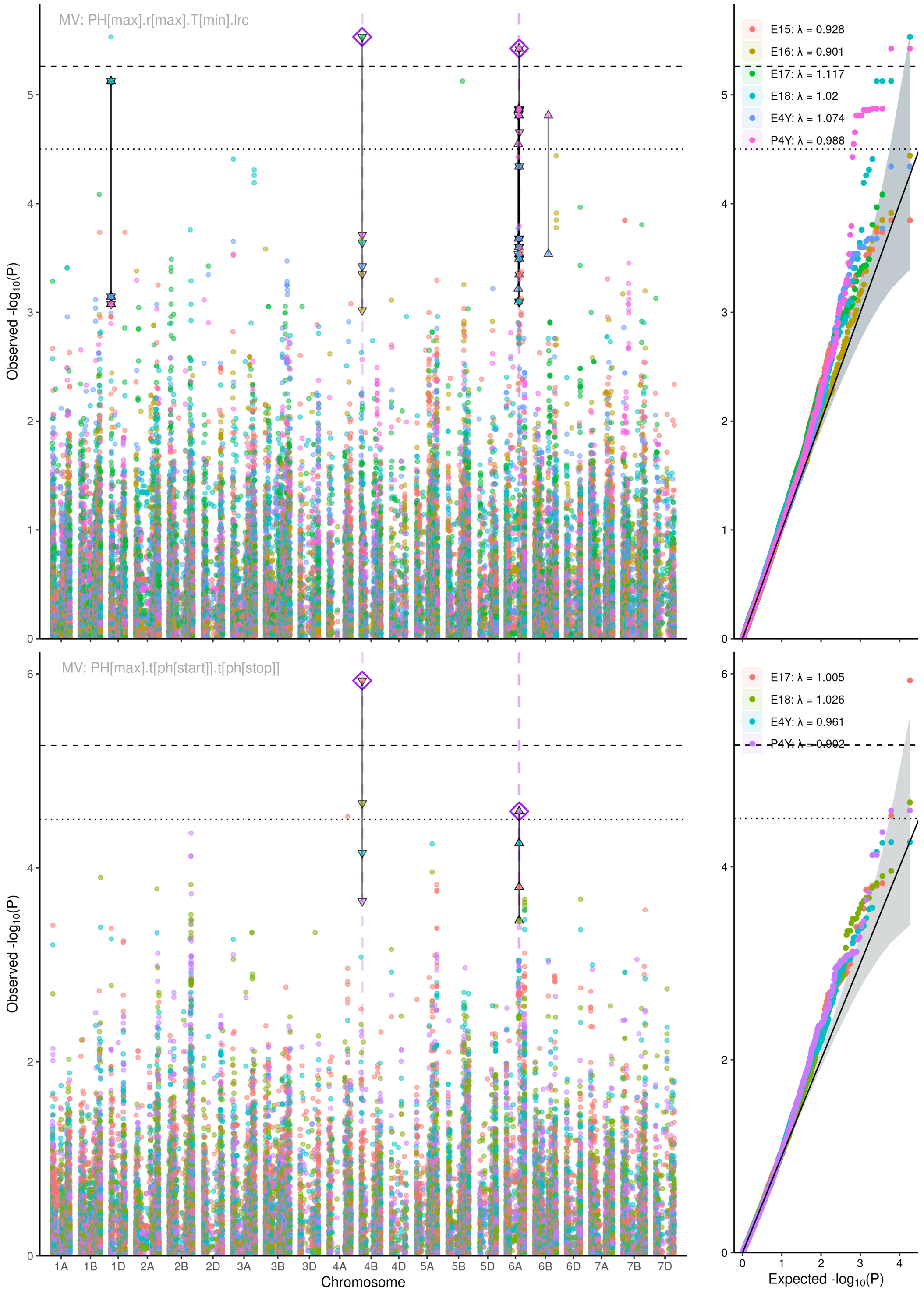

Figure S12: Manhattan plots and quantile–quantile plots depicting the multi-variate GWAS results for: (a) the temperature response traits $r_{\max}$,$T_{\min}$,lrc; (b) the temperature response traits and $PH_{\max}$, (c) all traits ($r_{\max}$,$T_{\min}$,lrc, $PH_{\max}$, $t_{\mathrm{PH}_{\mathrm{stop}}}$ and $t_{\mathrm{PH}_{\mathrm{start}}}$); (d) $PH_{\max}$ combined with the phenology traits $t_{\mathrm{PH}_{\mathrm{stop}}}$ and $t_{\mathrm{PH}_{\mathrm{start}}}$; and phenology traits $t_{\mathrm{PH}_{\mathrm{stop}}}$ and $t_{\mathrm{PH}_{\mathrm{start}}}$. In each plot, the results are given for each year (see color legend) including the respective test statistic inflation factor λ. MTAs detected across multiple models irrespective of the yearsite are indicated by purple rhombs and dashed vertical lines. These include also non-significant MTAs above the visually determined threshold of LOD > 4.5. The dashed horizontal lines indicate the Bonferroni corrected significance threshold for $\alpha = 0.05$ (i.e. LOD > 5.43).
